# Profiling of long non-coding RNAs identifies LINC00958 and LINC01296 as candidate oncogenes in bladder cancer

**DOI:** 10.1038/s41598-017-00327-0

**Published:** 2017-03-24

**Authors:** Anna Katharina Seitz, Lise Lotte Christensen, Emil Christensen, Kasper Faarkrog, Marie Stampe Ostenfeld, Jakob Hedegaard, Iver Nordentoft, Morten Muhlig Nielsen, Johan Palmfeldt, Michelle Thomson, Michael Theis Solgaard Jensen, Roman Nawroth, Tobias Maurer, Torben Falck Ørntoft, Jørgen Bjerggaard Jensen, Christian Kroun Damgaard, Lars Dyrskjøt

**Affiliations:** 1 Department of Molecular Medicine, Aarhus University Hospital, University of Aarhus, Aarhus, Denmark; 2 Department of Urology, Klinikum rechts der Isar, Technical University Munich, Munich, Germany; 3 Research unit for Molecular Medicine, Department of Clinical Medicine, Aarhus University Hospital, University of Aarhus, Aarhus, Denmark; 4 Department of Molecular Biology and Genetics - Genome expression, stability and technology, University of Aarhus, Aarhus, Denmark; 5 Department of Urology, Aarhus University Hospital, University of Aarhus, Aarhus, Denmark

## Abstract

Aberrant expression of long non-coding RNAs (lncRNAs) has been regarded as a critical component in bladder cancer (BC) and lncRNAs have been associated with BC development and progression although their overall expression and functional significance is still unclear. The aim of our study was to identify novel lncRNAs with a functional role in BC carcinogenesis. RNA-sequencing was used to identify aberrantly expressed lncRNAs in 8 normal and 72 BC samples. We identified 89 lncRNAs that were significantly dys-regulated in BC. Five lncRNAs; LINC00958, LINC01296, LINC00355, LNC-CMC1-1 and LNC-ALX1-2 were selected for further analyses. Silencing of LINC00958 or LINC01296 *in vitro* reduced both cell viability and migration. Knock-down of LINC00958 also affected invasion and resistance to anoikis. These cellular effects could be linked to direct/indirect regulation of protein coding mRNAs involved in cell death/survival, proliferation and cellular movement. Finally, we showed that LINC00958 binds proteins involved in regulation and initiation of translation and in post-transcriptional modification of RNA, including Metadherin, which has previously been associated with BC. Our analyses identified novel lncRNAs in BC that likely act as oncogenic drivers contributing to an aggressive cancerous phenotype likely through interaction with proteins involved in initiation of translation and/or post-transcriptional modification of RNA.

## Introduction

The human genome contains ~20,000 protein coding genes, covering less than 2% of the total genome sequence^1^. However, it has recently become clear that more than 80% of the genome is actively transcribed and accordingly, the vast majority of the transcribed genes are non-protein coding^2^. Apart from classical house-keeping non-coding RNAs (ncRNAs) including ribosomal RNA (rRNA), small nucleolar RNAs (snoRNAs), small nuclear RNA (snRNA), transfer RNAs (tRNA) and well-studied microRNAs (miRNAs), the major part of this group is represented by long non-coding RNAs (lncRNAs). LncRNAs are more than 200 nucleotides in length and their expression is controlled by both transcriptional and epigenetic factors. Like protein coding RNAs, many lncRNAs are generated by RNA polymerase II and undergo post-transcriptional modifications^3, 4^. In contrast, lncRNAs lack evolutionary conservation across species and show low but highly specific expression patterns^1, 5, 6^. Recently, high throughput profiling technologies have revealed thousands of lncRNAs that are differentially expressed between various cancers and their corresponding normal tissues^7^. Furthermore, the dysregulation of lncRNAs has been found to play important roles in the development of cancer and in several other diseases^8–10^. LncRNAs have been implicated in various fundamental cellular processes such as growth, chromosomal imprinting, dosage compensation, cell development, differentiation and pluripotency as well as DNA-damage^11–13^. A widely proposed functional model is based on lncRNAs ability to form complexes with e.g. proteins, mRNA and miRNAs. Hence, by exploiting their secondary and tertiary structure lncRNAs serve as guides, decoys or scaffolds thereby regulating gene transcription, mRNA stability or translation (reviewed in refs 6, 8, 14). However, although widely studied, the mechanisms of action of most lncRNA remain elusive.

Bladder cancer (BC) is one of the most common cancers worldwide and it ranks fifth among cancers in men in the Western countries. The molecular pathology of BC has been studied extensively. Genomic polymorphism and instability, chromosomal aberrations, epigenetic and genetic alterations characterize its molecular landscape and contribute to BC pathogenesis^15^. However, less is known about the functional significance of lncRNAs in BC pathogenesis (reviewed in refs 16, 17). Some of the most extensively studied lncRNAs in BC are H19, MALAT1, UCA1, HOTAIR and MEG3. H19, was found to be up-regulated in two independent BC patient cohorts, and increased expression levels were significantly correlated with either high risk of early tumor recurrence or risk of metastasis^18, 19^. Similar findings were published for MALAT1 and HOTAIR^20–25^. For UCA1, utility as urine marker for detection of primary or recurrent BC has been suggested, and a significant association with tumor grade and stage has also been observed^26, 27^. LncRNAs acting as tumor suppressors in BC are even less explored. Recently, DRAIC expression was shown to predict good prognosis in different cancers including BC^28^. Furthermore, knock-down of MEG3, a known activator of p53, was found to increase the proliferation of bladder cells^29, 30^.

Here we identified 89 significantly dysregulated lncRNAs in BC. The most significantly up-regulated lncRNAs were further characterized *in vitro*. Using loss of function studies we showed that LINC00958 and LINC01296 exhibit oncogenic functions in BC cell lines by regulating mRNAs associated with pathways related to cell death/survival and cellular movement. RNA pull-down and RNA immunoprecipitation (RIP) showed that LINC00958 interacts with proteins related to regulation and initiation of translation and/or post-transcriptional modification of RNA, consistent with potential oncogenic functions in BC.

## Results

### Delineation of aberrantly expressed lncRNAs in BC

**Figure 1:**
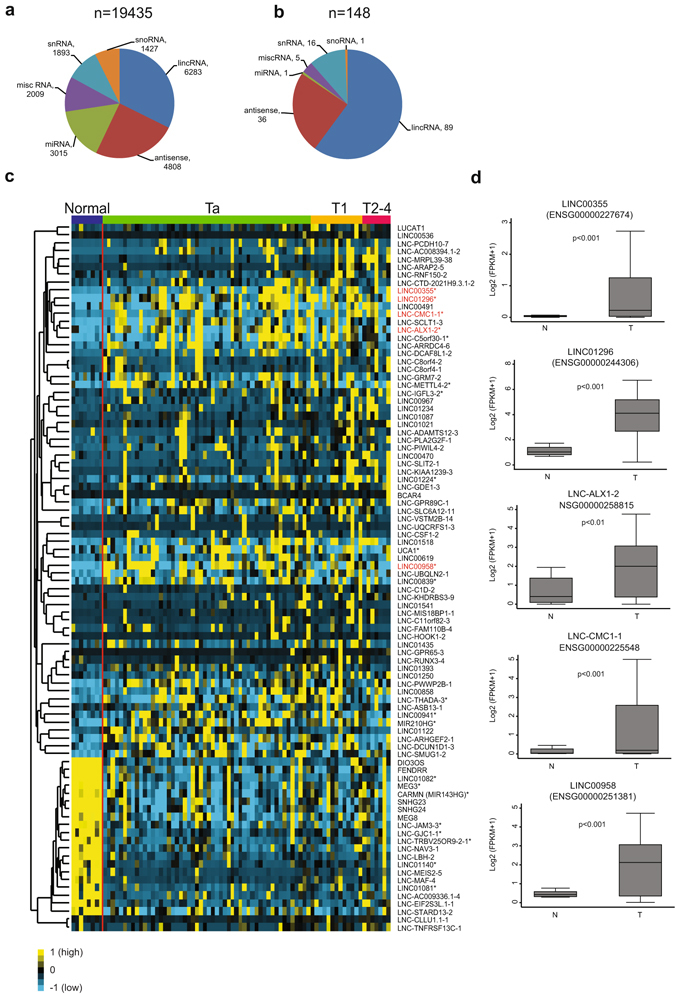
Non-protein coding RNA expression in bladder cancer. (**a**) Overall biotype distribution of non-protein coding transcript (i.e. lincRNAs, antisense RNAs, miRNAs, snoRNAs, snRNAs and miscRNAs) identified using Cufflinks and Cuffmerge and annotated according to Gencode v19. (**b**) Biotype distribution of the significantly differentially expressed non-protein coding genes using CuffDiff analysis. (**c**) Heat map of 89 significantly differentially expressed lincRNAs between normal urothelium and BC. Rows represent individual lincRNAs, columns represent individual tissue samples (sorted according to tumor stage as indicated). Yellow indicates up-regulation and blue indicates down-regulation. Genes are mean centered (black color) and normalized. LincRNAs selected for functional characterization are highlighted in red. *LncRNAs up/down regulated in the TCGA dataset (FC_log2_ > 0.15 or <−0.15, p < 0.01). (**d**) The expression of the five selected lincRNAs in normal (n = 8) urothelium compared to BCs (n = 72). Significance was tested using Student’s *t*-test. FPKM: Fragment Per Kilobase of exon per Million mapped reads.

RNA from 72 bladder tumors (52 pTa, 13 pT1 and 7 pT2-4) and 8 normal samples were subjected to RNA sequencing (RNA-seq)^31^. In total, 19435 non-protein coding transcripts (long intergenic non-coding RNAs (lincRNAs), antisense RNAs, miRNAs, snoRNAs, snRNAs and miscellaneous RNA (miscRNAs)) were identified. The most frequently annotated category among non-coding transcripts was lincRNAs followed by antisense lncRNA (Fig. 1a). Using CuffDiff2^32^ we identified 89 significantly dysregulated lincRNAs in BC when compared to normal bladder tissue (p < 0.01; multiple testing correction; Fig. 1b,c).

We focused our attention on lincRNAs up-regulated in BC and selected five of the most differentially expressed for detailed functional characterization (Fig. 1c, (shown in red) d). All five candidates were shown to have low predicted protein-coding scores using CPAT (Coding-Potential Assessment Tool), confirming their classification as noncoding transcripts (Supplementary Table S1)^33^. Application of PhastCon 46-way conservation track indicated conserved elements across vertebrate species exclusively for LINC00958 and LINC01296, suggesting a possible evolutionarily conserved function for these two lincRNAs (Supplementary Table S1). The differential expression of the lncRNAs was validated using data from The Human Cancer Genome Atlas (TCGA) provided by The Atlas of NcRNA In Cancer (TANRIC)^34^. Overall 22/89 lncRNAs were found to be up/down regulated in the TCGA BC samples (Fig. 1c and Supplementary Fig. S1). TCGA data was also used to analyze the expression of our five candidate lincRNAs in eight additional cancer types originating from seven different tissues (Supplementary Fig. S1). All five lincRNAs were significantly up-regulated in five or more of the analyzed cancers. Additionally, LINC00958 was significantly down-regulated in breast and prostate cancer.

### *In-vitro* model for functional characterization

RNA-seq of a panel of tumorigenic (RT4, T24, HT1197, HT1376, J82, SLT4, FL3, UMUC3 and LUL2) and immortalized (NHUtert and HCV29) urothelial cell lines was used to identify cell lines with high expression of the key candidates. All five lincRNAs were expressed at various levels in the 11 bladder cell lines (Supplementary Fig. S2). We selected FL3 (a metastatic isogenic derivate of the non-metastatic parental cell line T24) to investigate the functional impact of LINC00958 and LINC01296, whereas the non-metastatic HT1197 and HT1376 cells lines were selected for characterizing LINC00355, LNC-CMC1-1 and LNC-ALX1-2. All selected cell lines were derived from muscle invasive transitional cell carcinoma. RT4 cells were included to have a representative of a cell line derived from a non-invasive papillary tumor.

### Subcellular localization and knock-down of key candidate transcripts

**Figure 2:**
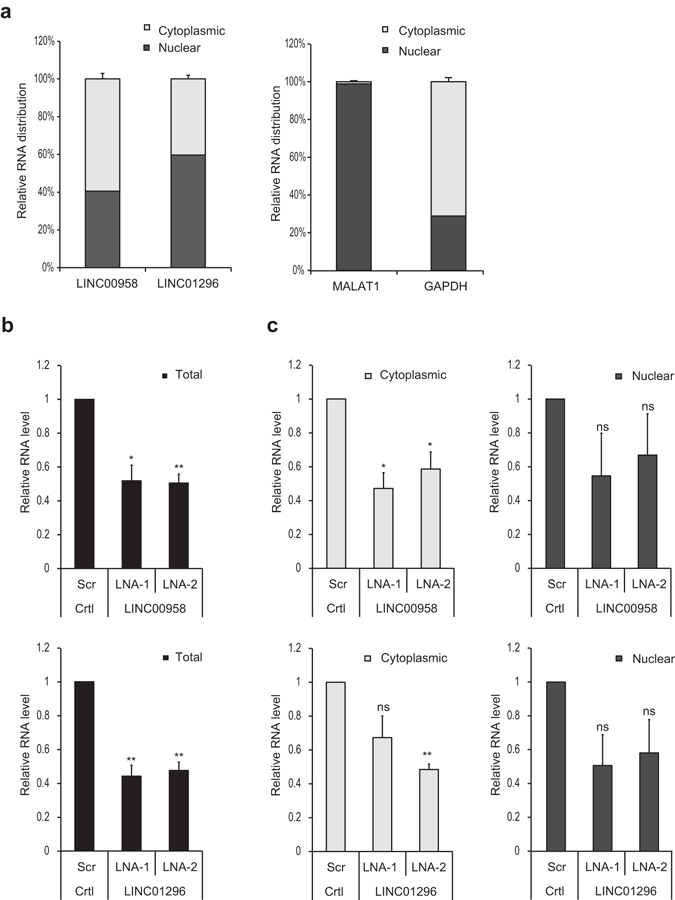
Subcellular distribution and knock-down efficiency of LINC00958 and LINC01296 in FL3 cells. (**a**) Percentage of total RNA found in the nuclear and cytoplasmic fraction in FL3 cells determined by RT-qPCR. Left panel: lincRNA-candidates. Right panel: nuclear (MALAT1) and cytoplasmic (GAPDH) markers for fraction purity. (**b** and **c**) FL3 cells were transfected with the indicated LNA longRNA GapmeR (50 nM) followed by total (**b**) or fractionated (**c**) RNA extraction 48 hours post-transfection. Knock-down-efficiency of lncRNA-candidates was analyzed by RT-qPCR. Relative total RNA expression levels were normalized to GAPDH and compared to Scr. Relative subcellular RNA expression levels were normalized to Scr. Columns represent the average of two independent experiment measured in triplicates. Bars: standard deviation (SD), ns: not significant and *p < 0.05 and **p < 0.01.

**Figure 3:**
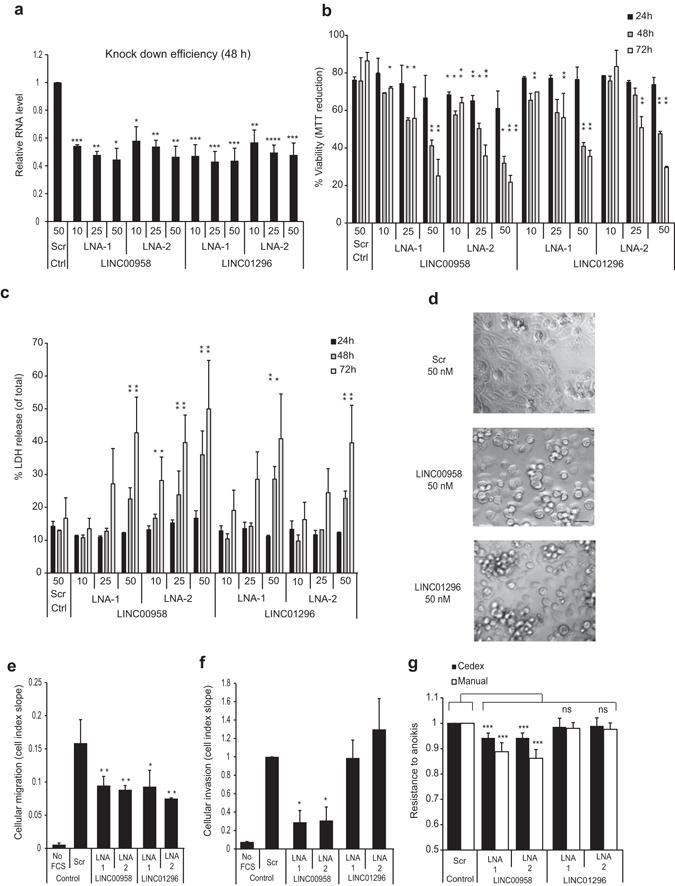
Functional effects of LINC00958 or LINC01296 knock-down in FL3 cells. FL3 cells were transfected either with a non-targeting LNA^TM^GapmeR (Scr) or 2 different LNA^TM^GapmeR separately targeting LINC00958 or LINC01296 (LNA-1 and LNA-2) at the indicated concentrations (10, 25 or 50 nM). (**a**) Dose-dependent knock-down efficiency was verified by RT-qPCR 48 hours post-transfection. Results were normalized to Scr. (**b**) Dose-dependent viability was assessed by MTT reduction 24, 48 and 72 h post-transfection and expressed as percentage compared to viability of untransfected cells. (**c**) Dose-dependent cell death was determined by the LDH release assay 24, 48 and 72 h post-transfection and depicted as percentage of released LDH out of total cellular LDH. (**d**) Cell morphology was examined upon candidate knock-down by phase contrast microscopy 72 h post-transfection (25x magnification, scale bar 40 μm). Cellular migration (**e**) and invasion (**f**) of transfected FL3 cells (25 nM LNA^TM^GapmeR). (**g**) Anoikis resistance upon candidate knock-down (25 nM LNA^TM^GapmeR). Cell viability was assessed by both automatic and manual cell counting. Results were normalized to Scr. FCS: Fetal calf Serum. Columns in (**b**,**c** and **g**) represents the average of two or three independent experiments, each measured in three to five replicates. Bars: SD and *p < 0.05, **p < 0.01, ***p < 0.001 and ****p < 0.0001.

RNA from nuclear and cytoplasmic fractions of FL3, RT4, HT1376 and HT1197 cells was used to determine the subcellular localization of our five candidates. RT-qPCR analysis revealed a roughly equal distribution of LINC00958 and LINC01296 in both subcellular compartments of FL3 cells (Fig. 2a), while high nuclear enrichment was found for LINC00355, LNC-CMC1-1 andLNC-ALX1-2 in HT1376 and HT1197 cells (Supplementary Fig. S3A). Interestingly, in the non-metastatic RT4 cell line derived from a papillary tumor, LINC00958 and LINC01296 expression was also predominantly nuclear enriched (Supplementary Fig. S4A). Successful isolation of pure nuclear and cytoplasmic fractions was confirmed by two marker RNA molecules with known nuclear (MALAT1) or cytoplasmic (GAPDH) enrichment (Fig. 2a and Supplementary Figs S3A and S4A). Using two different LNA (Locked Nucleic Acid)™ longRNA GapmeRs we observed ~50% total knock-down for LINC00958 and LINC01296 48 h post transfection when compared to Scramble (Scr)- transfected control cells (Fig. 2b). Comparable knock-down efficiencies were observed in the nucleus and cytoplasm for both candidates (Fig. 2c and Fig. S5A,B). The knock-down efficiency of the nuclear enriched candidates (LINC00355, LNC-CMC1-1 and LNC-ALX1-2) was 50–80% (Supplementary Fig. S3B). Dose-dependent knock-down efficiency was evaluated in the selected candidate-specific cell lines demonstrating most sufficient knock-down effects for 25 and 50 nM of LNA longRNA^TM^ GapmeR for all candidates (Fig. 3a and Supplementary Fig. S3B).

### LINC00958 and LINC01296 show oncogenic properties in BC cells *in vitro*

Next, we investigated the potential functional role of our key candidates in BC cells. Silencing of LINC00958 or LINC01296 resulted in a time and dose dependent reduction in cell viability (Fig. 3b). In contrast, knock-down of LINC00355, LNC-CMC1-1 and LNC-ALX1-2 did not affect cell viability (Supplementary Fig. S3C) and hence we focused on LINC00958 and LINC01296 in the subsequent analyses. To clarify whether reduction of cell density caused by LINC00958 and LINC01296 knock-down was related to cell death, we analyzed plasma membrane integrity upon LINC00958 and LINC01296 silencing using a Lactate dehydrogenase (LDH)-assay. Consistent with the effect observed in the viability analyses, we found a dose/time- dependent release of LDH with significant differences 48 hours post-transfection when compared to control cells (Fig. 3c). However, the LDH-assay does not distinguish between different pathways of programmed cell death. To address this point we performed cellular morphology studies since it is commonly known that different programmed cell death modes can be defined on the basis of cellular morphology^35^. LINC00958 or LINC01296 depleted FL3 cells displayed cell rounding and shrinkage, plasma membrane blebbing followed by subsequent detachment of the cells reflecting classical apoptosis (Fig. 3d). We next assessed the effects of LINC00958 and LINC01296 depletion on cell migration and invasion, both crucial steps during cancer progression and metastatic colonization. Knock-down of LINC00958 or LINC01296 significantly inhibited migration of the metastatic FL3 cell line (Fig. 3e) but only LINC00958-depleted cells demonstrated reduced invasion (Fig. 3f). Since LINC00958 and LINC01296 silencing also affected viability we cannot rule out that the reduced number of migrating cells could also be due to a reduction in the number of viable cells. However, the effect on viability was most pronounced at later time-points (≥48 h) (Fig. 3b,c). Accordingly, the reduction in migration/invasion at the early time points (≤45 h) used to calculate the cell index slope is most likely due to an actual decreased migration ability of LNA-treated cells (Supplementary Fig. S6A,B). During the metastatic cascade, cancer cells escape anoikis when arrested in circulation^36^. In line with this we have previously shown high anoikis resistance of the metastatic FL3 cell line^37^. Here, knock-down of LINC00958 but not of LINC01296 significantly attenuated anoikis resistance of FL3 cells (Fig. 3g). To corroborate the above findings and to exclude cell-line specific effects, functional consequences of LINC00958 and LINC01296 knock-down was also investigated in the RT4 cell line in which we observed similar anti-proliferative and cell-death-inducing effects upon LINC00958 and LINC01296 knock-down (Supplementary Fig. S4B–D). As previously reported it was not possible to carry out migration and invasion assays for RT4 cells, a phenomenon that may be attributed to its derivation from a noninvasive tumor^38^. Altogether, the results indicate that LINC00958 and LINC01296 may exhibit oncogenic properties by promoting proliferation, migration and invasion (only LINC00958) and hence may play a role during the metastatic process of BC cells.

### Transcriptional profiling and pathway analysis in LINC00958 and LINC01296 depleted FL3 cells

**Figure 4:**
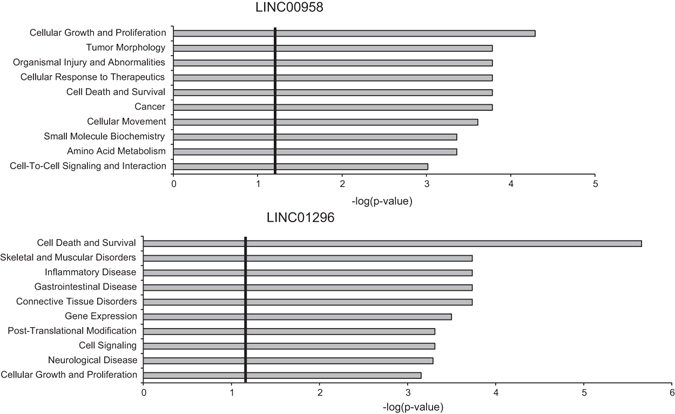
Ingenuity Pathway Analysis. The most significantly biological functions and diseases associated with altered intracellular levels of LINC00958 or LINC01296 in FL3 cells 24 h post transfection are shown. Only RNAs demonstrating log2 ratios <−0.6 or >0.6, when comparing cells transfected with LNA^TM^GapmeR (Scr) to cells transfected with LNA^TM^GapmeRs targeting either LINC00958 or LINC01296 (LNA-1 and LNA-2), were included in the analysis (LINC00958: n = 1933 genes and LINC01296: n = 915 genes). Threshold value; p = 0.05. The analyses were based on knowledge from all species, all tissues, and all data sources.

To further investigate genome wide molecular consequences of LINC00958 and LINC01296 knock-down in FL3 cells, transcriptome profiling was carried out using microarray analysis 24 h post-transfection. We identified 1933 significantly dysregulated transcripts (665 up- and 1268 down-regulated) upon LINC00958 knock-down and 915 significantly dysregulated transcript (215 up- and 705 down-regulated) when LINC01296 was depleted (FC_(log2)_ < −0.6 or >0.6). Ingenuity Pathway Analysis demonstrated that both LINC00958 and LINC01296 knock-down changed the expression of genes associated with molecular and cellular functions related to cell death/survival and cellular growth/proliferation (*p* < 0.05) in agreement with the observed negative effect on BC cell viability and induction of cell death (Fig. 4). Furthermore LINC00958 knock-down also had a negative effect on cellular functions related to cellular movement (*p* < 0.05) correlating with the reduced migratory and invasive potential observed as a result of LINC00958 knock-down. To investigate whether some of the altered genes were affected in clinical samples; we set out to explore their expression pattern in the 72 BC tissues samples used for lncRNA expression analysis. Out of 1268 genes down-regulated upon LINC00958 knock-down 44 genes were positively correlated to the expression of LINC00958 in tumor samples (Spearman’s ρ ≥ 0.4 and *p* < 0.01 (Bonferroni corrected)) (Supplementary Table S2). The positive correlation with LINC00958 was confirmed for ITGA3, SMARCA1 and AIG1 in an independent cohort of 476 BC samples (data not shown)^39^. Oncogenic roles have previously been assigned to both ITGA3 and SMARCA1 whereas the tumorigenic role of AIG1 has not been described in detail^40–42^. Similarly, 27 of 705 genes down-regulated upon LINC01296 knock-down were positively correlated to the expression of LINC01296 in tumor samples (Spearman’s ρ ≥ 0.4 and *p* < 0.01 (Bonferroni corrected)) (Supplementary Table S3). However, none of these correlations could be confirmed in the independent data set.

LincRNAs have previously been shown to regulate the expression of neighboring genes. The genomic region surrounding LINC00958 and LINC01296 harbor 4 genes (TEAD1 and RASSF10 (LINC00958) and LINC01297 and POTEM (LINC01296)) within 100 kbs up- or downstream of the lincRNA locus (Supplementary Fig. S7A and B). The expression of LINC01296 was found to be significantly correlated to both LINC01297 (Spearman’s ρ 0.60 and p < 0.0001) and POTEM (Spearman’s ρ 0.48 and p < 0.0001). If LINC01296 was involved in regulating the expression of either LINC01297 and/or POTEM one would expect the expression of these genes to be affected by the knock-down of LINC01296. Unfortunately, the expression of LINC01297 and POTEM was not analyzed by Affymetrix GeneChip® Human Gene 2.0 ST array hence their potential regulation by LINC01296 was not analyzed further.

### Identification of endogenous proteins associated with LINC00958

**Figure 5:**
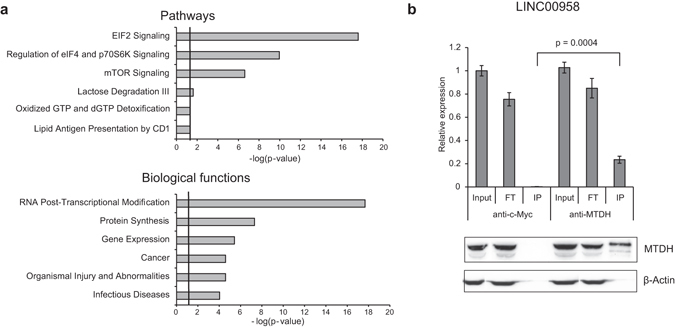
*In vitro* pull-down and mass spectrometry analysis identified LINC00958 interacting proteins. (**a**) Ingenuity Pathway Analysis. The most significantly pathways and biological functions associated with proteins binding biotin-tagged LINC00958. Only proteins demonstrating FCs ≥ 3, when comparing biotin-tagged LINC00958 to controls were included in the analysis (n = 330). Threshold value; p = 0.05. The analyses were based on knowledge from all species, all tissues, and all data sources. (**b**) Anti-MTDH RIP using FL3 cell lysates. Detection of endogenous LINC00958 was performed using RT-qPCR (upper panel). The RT-qPCR was performed in triplicates. The experiment was repeated twice and the result of one representative experiment ±SD is shown. IP with a c-Myc antibody was included as negative control. Western blotting analysis (lower panel) was used to evaluate IP of MTDH in the analyzed samples. Loading control input samples: β-actin. FT: flow-through and IP: immunoprecipitation.

Overall the most pronounced phenotypic effects in BC cells were shown for LINC00958. Therefore, we set out to identify LINC00958 protein interaction partners to further elucidate the mechanisms of action of LINC00958. *In vitro* transcribed biotin-tagged LINC00958 RNA was used to pull-down endogenous proteins. LINC00958 RNA or control RNAs was captured by magnetic streptavidin beads. Using Northern blotting we showed that the integrity and relative pull-down efficiency of the LINC00958 and the control RNAs was high and at comparable levels (Supplementary Fig. S8A). The streptavidin captured RNA (LINC00958 and controls) was incubated with FL3 cell lysates followed by mass spectrometry. Pull-down of significant amount of protein was demonstrated specifically for biotinylated RNA (Supplementary Fig. S8B). The mass spectrometric analysis yielded 330 proteins that were significantly enriched using the LINC00958 RNA as bait compared to control RNAs (FC ≥ 3) (Supplementary File S1 and Supplementary Table S6). Many of these proteins were involved in the regulation and initiation of translation and in post-transcriptional modifications of RNA (Fig. 5a and Supplementary Tables S4 and S5). Interestingly, Metadherin (MTDH) (also referred to as AEG-1 and “Protein LYRIC”), which has previously been shown to be up-regulated in BC and associated with BC progression^43, 44^, was significantly associated with LINC00958. Using Western blotting, we confirmed the physical association between MTDH and LINC00958 in the pull-down samples. The Western blotting showed no interaction between MTDH and the control RNAs ciRS-7 and ZFAS RNA. Finally, the unrelated protein TIA-1 was not captured by any of the RNAs (Supplementary Fig. S8C). Furthermore, RPIseq (*in silico* RNA-Protein Interaction Prediction) resulted in interaction probabilities ranging between 0.66 and 0.80 indicating that MTDH and LINC00958 are likely to interact^45^. Importantly, using anti-MTDH or control anti-Myc antibodies (negative control) we readily validated the endogenous MTDH-LINC00958 interaction in reciprocal RIP experiments using FL3 lysates (Fig. 5b upper panel).

## Discussion

Accumulating evidence documents that non-protein coding genes play important roles in BC development and progression. This has been shown in studies of e.g. H19, MALAT1, UCA1 and MEG3 (reviewed in refs 16, 17). In a recent study by our group we delineated the non-coding gene expression landscape in 460 non-muscle invasive bladder tumors using RNA-seq^46^. In addition, a subgroup with basal-like characteristics was identified that exhibited remarkably elevated levels of lncRNA expression compared to expression of protein coding genes. Here we compared BC to normal urothelial samples using RNA-seq in order to identify non-coding genes with tumor suppressor or oncogenic functions. We identified 89 significantly dysregulated lincRNAs including the known BC lncRNAs MEG3 and UCA1^27, 30^. We selected five significantly up-regulated lincRNAs that had not previously been characterized in BC, for functional studies.

Knowledge about cellular localization of lncRNAs is needed to unravel biological functions. We showed that LINC00958 and LINC01296 localized to both the nucleus and the cytoplasm while high nuclear enrichment was found for LINC00355, LNC-CMC1-1 andLNC-ALX1-2. Nuclear lncRNAs may play roles in chromatin interactions, transcriptional regulation and RNA processing whereas cytoplasmic lncRNAs may be involved in post-transcriptional processes such as mRNA stability or translation, transport and subcellular localization of proteins^14^. Although initial analyses by Derrien *et al.* demonstrated nuclear enrichment of lncRNAs, more recent studies have shown that the majority of lncRNAs are mostly found in the cytoplasm where they often associate with ribosomes, even though they are likely not actively translated^1, 47, 48^. Using *in vitro* analyses we showed that LINC00958 and LINC01296 silencing reduced cell growth and migration in BC cells. Moreover, knock-down of LINC00958 also reduced invasion and resistance to anoikis, both crucial steps during cancer progression and metastatic colonization. These phenotypes indicate that LINC00958 and to a lesser extent LINC01296 act as oncogenes by promoting proliferation and different steps of the metastatic process in BC cells. In agreement with the phenotypic data, pathway analysis revealed that LINC00958 and LINC01296 knock-down primarily affected molecular and cellular functions related to cell death/survival and cellular movement. Interestingly, the mRNAs of ITGA3, SMARCA1 and AIG1, which were down-regulated as a result of LINC00958 silencing, were also positively correlated with the expression of LINC00958 in clinical samples. The inhibitory effect on ITGA3 and SMARCA1 is in line with the observed phenotypic effects since silencing of ITGA3 has previously been shown to diminish the migratory capacity of colorectal cancer cells, whereas growth inhibition and increased apoptosis have been reported in breast cancer cell lines after SMARCA1 knock-down^40–42^.

Several studies have suggested that nuclear lncRNAs exert their function through binding to proteins or protein complexes such as the histone modification complex PRC2, which functions as a transcriptional repressor (reviewed in ref. 4). The lncRNA HOTAIR has been shown to promote cancer progression through its interaction with PRC2 proteins^49^. Additionally, the cytoplasmic lincRNA-p21 has been shown to be involved post-transcriptional inhibition of translation by binding the translational repressors Rck^50^. Here we found that LINC00958 bound proteins involved in regulation and initiation of translation and in post-transcriptional modification of RNA and provided evidence that LINC00958 associates with MTDH. The RIP analysis performed in the present study identifies proteins that associate either directly or indirectly with LINC00958. However, interaction probabilities calculated using RPIseq indicated that MTDH and LINC00958 are likely to interact directly. Most interestingly, silencing of MTDH in BC and endometrial cancer cell lines resulted in reduced cellular viability and induction of apoptotic death similar to the phenotypes observed upon knockdown of LINC00958 in the present study^51, 52^. Furthermore, cytoplasmic MTDH has been demonstrated to act as an RNA binding protein and to negatively regulate the translation of multiple mRNAs^52^. We speculate that the LINC00958/MTDH complex may be involved in regulating the translation of mRNAs, a hypothesis that is supported by the binding of other proteins related to translational regulation in the LINC00958 RIP analysis. Alternatively, LINC00958 may also be involved in controlling the cellular localization of MTDH. A substantial number of lncRNAs are known to regulate trafficking of proteins e.g. the lncRNA NRON, which has been found to regulate the nuclear localization of NFAT (Nucleus Factor of Activated T-cells)^53^. Previous studies have identified the MTDH protein in the nucleus as well as the cytoplasm depending on the examined cell types^54^. However, in BC the up-regulated MTDH protein is primarily found in the cytoplasm^44^. Nevertheless further analyses are required to elucidate the mechanisms of action and functional role of the MTDH-LINC00958 association (as well as the other associations identified in the RIP analysis).

In conclusion, we identified a large number of lncRNAs significantly dysregulated in BC compared to normal urothelium. The lincRNAs LINC00958 and LINC01296 were significantly up-regulated and displayed phenotypic functions indicative of oncogenic properties in BC. The oncogenic function of LINC00958 is likely associated with RNA binding proteins including MTDH by yet undefined mechanisms.

## Materials and Methods

### Clinical samples, RNA extraction, cDNA synthesis and RNA-seq

The use of the human tissue samples was approved by the Danish national scientific ethical committee (file #1300174), and informed written consent was obtained from all patients. The methods in the study were carried out in accordance with the approved guidelines and regulations. The cohort consisted of 72 (52 Ta, 13 T1 and 7 T2-4) histologically verified bladder tumors from patients treated at the Department of Urology, Aarhus University Hospital (1995–2011) and 8 biopsies of normal bladder mucosa from patients without any BC history. Detailed information regarding the patient cohort, tissues samples, RNA extraction, cDNA synthesis and RNA-seq is found in ref. 31. The Gencode v15 without pseudogenes was used for annotation of the detected transcripts in the RNAseq analyses. The differentially expressed lincRNAs were named according to LNCipedia 4.0, which uses the official gene symbols for lncRNAs from the Hugo Gene Nomenclature Committee (HGNC). Expression of selected lincRNAs was validated in BC samples and in samples from additional cancers using datasets from TCGA (http://cancergenome.nih.gov/) provided by TANRIC. The miRNeasy mini kit (Qiagen) was used for transfected cells and for the samples from the MTDH RIP analysis according to the manufacturer’s instructions.

### Cell lines

BC cell lines RT4, HT1376, HT1197, T24, J82, and UMUC3 (American Type Culture Collection (ATCC)), SLT4, FL3 and LUL2 (Prof. Dan Theodorescu, University of Colorado Cancer Center, Aurora, CO, USA) and immortalized human bladder epithelium (HCV29 (Prof. Jesper Zeuthen, Danish Cancer Society, Copenhagen, Denmark)) and hTERT-NHU (Prof. M.A. Knowles, University of Leeds, Leeds, United Kingdom) cells were used in the present study. Cell line authentication was confirmed using Cell-ID (Promega, Fitchburg, WI, USA). The cell lines were grown as described previously^37, 55, 56^ except for hTERT-NHU cells which were cultured in Keratinocyte-SFM (Life Technologies).

### Real-Time Quantitative Reverse Transcription PCR (RT-qPCR)

One µg RNA was reverse transcribed and used for SYBR^TM^ Green-based (Life Technologies, Carlsbad, CA, USA) RT-qPCR using Applied Biosystems® ViiA™ 7 Real-Time PCR System (Life Technologies). Primer sequences (Sigma Aldrich, St. Louis, MO, USA) are listed in Supplementary Materials. Raw C_T_ values were calculated with automatic baseline and threshold settings by the ViiA™ 7 Software 1.2.2 (Life Technologies). Relative expression levels were determined using either the standard curve method or the comparative C_T_ method (2^−ΔΔCT^ method). Except for the cell fractionation assays lncRNAs expression data were normalized to the endogenous reference gene GAPDH.

### LncRNA knock-down using LNA™ longRNA GapmeRs

LNA™ longRNA GapmeRs (antisense oligonucleotides (ASOs)) of 14–16 nucleotides) (Exiqon, Vedbaek, Denmark) are listed in Supplementary Materials. LNA™ longRNA GapmeRs or Scr control were reverse transfected at the indicated final concentrations and time points using Lipofectamine 2000 (Life Technologies) according to the manufacturer’s guidelines. Medium was changed 8 hours post transfection.

### Cell fractionation and isolation of RNA from BC cell lines

The Protein And RNA Isolation System (PARIS) (Ambion, Life Technologies) was used to partition the bladder cell lines into cytoplasmic and nuclear fractions followed by isolation of RNA from each fraction according to the manufacturer’s instructions. In parallel RNA was isolated from unfractionated cells (harvested with Qiazol) (Qiagen, Hilden, Germany) and purified using miRNeasy mini kit (Qiagen) according to the manufacturer’s instructions.

### Cell viability/death and Anoikis resistance analysis

Cell viability and death were analyzed by 3-(4,5-dimethylthiazol-2-yl)-2,5-diphenyltetrazolium bromide (MTT) reduction (Sigma-Aldrich, St. Louis, MO, USA) and Cytotoxicity Detection Kit (LDH-assay)(Roche, Basel, Switzerland) assays, essentially as described in ref. 57. The Anoikis resistance assay was performed with Corning Costar Ultra-Low attachment 24-well plates (Corning Life Science, Tewksbury, MA, USA). Briefly, 48 h post transfection, cells were plated and after another 24 h, cellular viability was assessed either manually by counting cells with loss of membrane integrity, or Trypan blue exclusion followed by automatic cell counting (Cedex XS Analyzer, Roche).

### Real-time migration and invasion

Real-time migration and invasion analysis were conducted essentially as described previously using CIM-16 plates with 8 µm pore sized membranes (Roche) and xCELLigence kinetic Systems (ACEA Biosciences, San Diego, CA)^37, 57^. The xCELLigence software (RTCA 1.2) was used to collect impedance measurements (Cell Index) every 10 min for up to 72 hours. Transfection of LNA™ longRNA GapmeRs was conducted 48 h prior to initiation of real-time migration or invasion monitoring. Quantification of migration and invasion was accomplished by calculation of the slope increment (rate of changes in cell index) of a certain period of time using the RTCA software.

### Microarray expression and Ingenuity Pathway analysis

For gene expression profiling upon knock-down of LINC00958 and LINC01296, RNA was extracted from LNA^TM^ GapmeR transfected cell lines (50 nM) 24 hours post-transfection, converted to cDNA, fragmented, labelled and hybridized onto a Affymetrix GeneChip® Human Gene 2.0 ST array according to the manufacturer’s protocol (Affymetrix, Santa Clara, CA, USA). Genechips were scanned with the Affymetrix 3000 7G Scanner. Normalization and background correction of the raw microarray data was done by Robust Multichip Average (RMA) algorithm using GeneSpring GX 11.5 software (Agilent, Santa Clara, CA, USA). A fold change (FC) cut-off of ±0.6 (log2) upon lncRNAs-candidate knock-down was applied to select dysregulated target genes for Ingenuity Pathway Analysis (Ingenuity Systems, www.ingenurity.com).

### Cloning of LINC00958 and biotin-LINC00958 RNA pull-down

Initially, the LINC00958 transcript variant ENST00000534477.1 was amplified from a pool of cDNA prepared from RNA isolated from human BC tissue samples and cloned into the pCR™4-TOPO® vector using the TOPO® TA Cloning® Kit for Sequencing (Life Technologies). Cloned sequences were validated by Sanger Sequencing. Primers used for cloning/sequencing are shown in the Supplementary Materials. Biotin-tagged LINC00958 RNA was prepared using T7 MEGAscript® transcription kit (Ambion, Life Technologies) according to the manufacturer’s protocol with the following exceptions: Biotin-14-CTP was included in the transcription reaction at 0.75 mM concentration (1:10 of regular CTP). One µg linearized and phenol/chloroform extracted pCR™4-TOPO® plasmid (Life Technologies) containing the LINC00958 gene was used as template for the transcription reaction. Transcribed RNA was purified by phenol/chloroform extraction, ethanol precipitated and the RNA pellet was re-suspended in nuclease free water. The following controls were included in the pull-down: Beads only, LINC00958 RNA without biotin, anti-sense LINC00958 RNA with biotin, ciRS7 (miR-7 sponge) and ZFAS1 (unrelated lncRNA). For each pull-down experiment 20 µl (bead volume) Pierce Magnetic Streptavidin beads® (Thermo Scientific, Waltham, MA, USA) were coupled to 30 µg *in vitro* synthesized LINC00958 or control RNA by incubation in 500 µl NET2 buffer [20 mM Tris-HCl pH 7.4, 150 mM NaCl, 0.1 mM EDTA (EthyleneDiamineTetraacetic Acid), 0.1%Triton X-100] supplemented with 40 U RiboLock® (Fermentas, Thermo Scientific) for 1 hr at 4 °C with gentle nutation. Beads were subsequently washed twice in 1.5 ml NET2 prior to incubation with cell lysates. Generelly, 90–95% of RNA was retained on the beads. FL3 cell lysis was carried out as follows: 8–10 × 10^6^ cells growing on a p15 plate, were washed twice in 10 ml ice-cold phosphate buffered saline (PBS), and lysed directlyon the plate in 1 ml hypotonic lysis buffer (10 mM Tris-HCl pH 7.4, 10 mM NaCl, 1 mM EDTA, 0.1% Triton-X100) which was supplemented with 1 pill Complete® (Roche) and 10 µl RiboLock (Fermentas, Thermo Scientific) per 10 ml lysis buffer. Cells were incubated for 5 mins on ice and scraped off using a rubber policeman, transferred to 1.5 ml tubes, supplemented with 30 µl 4 M NaCl (final 150 mM) and incubated for 5 mins on ice. Cleared lysate was prepared by centrifugation at 20000 × *g*, 4 °C, 15 mins and incubated with the LINC00958-coupled streptavidin beads for 1 hr, 4 °C with gentle nutation. Following protein capture beads were washed 4 times in 1.5 ml NET2. mRNA/protein (mRNPs) complexes were eluted by incubating in SDS load buffer at 90 °C for 4 mins. Eluates were subjected to SDS-PAGE electrophoresis (4–20% gradient gels) and gels were either stained using SilverQuest silver-staining kit (Invitrogen) or colloidal Coomassie blue. Bands were excised from a preparative Coomassie stained gel and prepared for mass spectrometry (MS) analyses as described below.

#### Northern blotting of RNA pull down

Validation of biotinylation, RNA pull-down and RNA integrity was conducted by Northern blotting of RNA from Input and IP fractions. After standard denaturing Agarose gel electrophoresis and blotting onto a Hybond N+ membrane, the RNA was x-linked (Stratalinker) according to previous protocols^59^ and the membrane blocked by Licor TBS blocking buffer for 1 hr. The biotinylated RNA was detected using 1:5000 dilution of Alexa-Flour594–Streptavidin in Licor TBS blocking buffer for 30 minures followed by 3 times washing in 50 ml TBS. Fluorescent RNA was detected and quantitated using a Licor OdysseyFc fluorescence scanner.

### Protein analysis by nano-LC-MS/MS

Interacting proteins were identified and quantified by nano-LC-MS/MS (Liquid Chromatography tandem Mass Spectrometry), as previously described^58^. Briefly, in-gel digestion was performed using trypsin and resulting peptides were C18-purified and analyzed on an EASY nanoLC (on a 25 cm C18 Pepmap column from Thermo) coupled to a Q Exactive Plus Hybrid Quadrupole-Orbitrap Mass Spectrometer (Thermo Scientific). Mass resolution was 70 000 and 17 500, for peptide and fragment scans, respectively. MaxQuant software version 1.5.2.8 was applied for protein identification (using 20 197 *Homo sapiens* sequences from Uniprot, August 2015), and label-free quantification by means of peptide peak areas^60^. False discovery rate (FDR) was set to 0.01 for both protein and peptide identification. Proteins were considered significant only if the protein was present in all 3 replicates of LINC00958 pull-down and displayed an overall fold enrichment over controls greater than 3.

### MTDH RNA-Immunopreciptiation

Cells were grown to ~70% confluency in DMEM including 10% Foetal Bovine Serum. The IP was carried out with rabbit polyclonal anti-MTDH (Abnova (H00092140-PW2), Dallas TX, USA) and performed as described previously^59^. MTDH IP was valuated using Western blotting analysis, according to standard procedures. Briefly, gel electrophoresis and protein transfer were performed using XCell SureLock Mini-Cell with Blot Module™ (Invitrogen, Life Technologies). Membranes were blocked and incubated for at least 16 hours at room temperature with mouse polyclonal anti-MTHD (Abnova (H00092140-PW2), Dallas TX, USA; dilution 1:500). Following incubation with goat anti-mouse horseradish peroxidase (HRP)-conjugated secondary antibody (Dako, Glostrup, DK; dilution 1:10.000) proteins were detected using ECL prime Western Blotting Reagent (GE Healthcare, Chalfont St. Giles, UK) and ChemiDoc-It Imaging System (UVP, Upland, CA, USA). The β-actin was visualized using HRP-conjugated mouse anti-β-actin (Abcam; dilution 1:10.000).

### Statistical analysis

Phenotypic effects in transfected cell lines and gene expression differences were analyzed by two-sided Student’s *t*-test (parametric testing) or Mann–Whitney *U* test (non-parametric testing) when appropriate. Unsupervised hierarchical cluster analysis was performed using Cluster 3.0 and Java tree-view software. Statistically significant *p*-values are indicated with asterisks as follows: *p < 0.05, **p < 0.01 and ***p < 0.001.

## Electronic supplementary material

Supplementary INFO

## References

[1] Derrien T (2012). The GENCODE v7 catalog of human long noncoding RNAs: analysis of their gene structure, evolution, and expression. Genome Res.

[2] Hangauer MJ, Vaughn IW, McManus MT (2013). Pervasive transcription of the human genome produces thousands of previously unidentified long intergenic noncoding RNAs. PLoS Genet.

[3] Guttman M (2009). Chromatin signature reveals over a thousand highly conserved large non-coding RNAs in mammals. Nature.

[4] Mercer TR, Mattick JS (2013). Structure and function of long noncoding RNAs in epigenetic regulation. Nat Struct Mol Biol.

[5] Cabili MN (2011). Integrative annotation of human large intergenic noncoding RNAs reveals global properties and specific subclasses. Genes Dev.

[6] Guttman M, Rinn JL (2012). Modular regulatory principles of large non-coding RNAs. Nature.

[7] Yan X (2015). Comprehensive Genomic Characterization of Long Non-coding RNAs across Human Cancers. Cancer Cell.

[8] Schmitt AM, Chang HY (2016). Long Noncoding RNAs in Cancer Pathways. Cancer Cell.

[9] Esteller M (2011). Non-coding RNAs in human disease. Nat Rev Genet.

[10] Spizzo R, Almeida MI, Colombatti A, Calin GA (2012). Long non-coding RNAs and cancer: a new frontier of translational research?. Oncogene.

[11] Geisler S, Coller J (2013). RNA in unexpected places: long non-coding RNA functions in diverse cellular contexts. Nat Rev Mol Cell Biol.

[12] Fatica A, Bozzoni I (2014). Long non-coding RNAs: new players in cell differentiation and development. Nat Rev Genet.

[13] Sanchez Y (2014). Genome-wide analysis of the human p53 transcriptional network unveils a lncRNA tumour suppressor signature. Nat Commun.

[14] Ulitsky I, Bartel D (2013). P. lincRNAs: genomics, evolution, and mechanisms. Cell.

[15] Knowles MA, Hurst CD (2015). Molecular biology of bladder cancer: new insights into pathogenesis and clinical diversity. Nat Rev Cancer.

[16] Martens-Uzunova ES (2014). Long noncoding RNA in prostate, bladder, and kidney cancer. Eur Urol.

[17] Zhang Q, Su M, Lu G, Wang J (2013). The complexity of bladder cancer: long noncoding RNAs are on the stage. Mol Cancer.

[18] Ariel I (2000). The imprinted H19 gene is a marker of early recurrence in human bladder carcinoma. Mol Pathol.

[19] Luo M (2013). Long non-coding RNA H19 increases bladder cancer metastasis by associating with EZH2 and inhibiting E-cadherin expression. Cancer Lett.

[20] Fan Y (2014). TGF-beta-induced upregulation of malat1 promotes bladder cancer metastasis by associating with suz12. Clin Cancer Res.

[21] Ying L (2012). Upregulated MALAT-1 contributes to bladder cancer cell migration by inducing epithelial-to-mesenchymal transition. Mol Biosyst.

[22] Han Y, Liu Y, Nie L, Gui Y, Cai Z (2013). Inducing cell proliferation inhibition, apoptosis, and motility reduction by silencing long noncoding ribonucleic acid metastasis-associated lung adenocarcinoma transcript 1 in urothelial carcinoma of the bladder. Urology.

[23] Yan TH (2014). Upregulation of the long noncoding RNA HOTAIR predicts recurrence in stage Ta/T1 bladder cancer. Tumour Biol.

[24] Heubach J (2015). The long noncoding RNA HOTAIR has tissue and cell type-dependent effects on HOX gene expression and phenotype of urothelial cancer cells. Mol Cancer.

[25] Martinez-Fernandez M (2015). Analysis of the Polycomb-related lncRNAs HOTAIR and ANRIL in bladder cancer. Clin Epigenetics.

[26] Wang XS (2006). Rapid identification of UCA1 as a very sensitive and specific unique marker for human bladder carcinoma. Clin Cancer Res.

[27] Srivastava AK (2014). Appraisal of diagnostic ability of UCA1 as a biomarker of carcinoma of the urinary bladder. Tumour Biol.

[28] Sakurai K, Reon BJ, Anaya J, Dutta A (2015). The lncRNA DRAIC/PCAT29 Locus Constitutes a Tumor-Suppressive Nexus. Mol Cancer Res.

[29] Zhou Y, Zhang X, Klibanski A (2012). MEG3 noncoding RNA: a tumor suppressor. J Mol Endocrinol.

[30] Ying L (2013). Downregulated MEG3 activates autophagy and increases cell proliferation in bladder cancer. Mol Biosyst.

[31] Lamy P (2016). Paired Exome Analysis Reveals Clonal Evolution and Potential Therapeutic Targets in Urothelial Carcinoma. Cancer Res.

[32] Trapnell C (2013). Differential analysis of gene regulation at transcript resolution with RNA-seq. Nat Biotechnol.

[33] Wang L (2013). CPAT: Coding-Potential Assessment Tool using an alignment-free logistic regression model. Nucleic Acids Res.

[34] Li J (2015). TANRIC: An Interactive Open Platform to Explore the Function of lncRNAs in Cancer. Cancer Res.

[35] Kroemer G (2009). Classification of cell death: recommendations of the Nomenclature Committee on Cell Death 2009. Cell Death Differ.

[36] Paoli P, Giannoni E, Chiarugi P (2013). Anoikis molecular pathways and its role in cancer progression. Biochim Biophys Acta.

[37] Ostenfeld MS (2014). Cellular disposal of miR23b by RAB27-dependent exosome release is linked to acquisition of metastatic properties. Cancer Res.

[38] Chen CL (2006). Valproic acid inhibits invasiveness in bladder cancer but not in prostate cancer cells. J Pharmacol Exp Ther.

[39] Hedegaard J (2016). Comprehensive Transcriptional Analysis of Early-Stage Urothelial Carcinoma. Cancer Cell.

[40] Bauer KM, Watts TN, Buechler S, Hummon AB (2014). Proteomic and functional investigation of the colon cancer relapse-associated genes NOX4 and ITGA3. J Proteome Res.

[41] Ye Y (2009). Inhibition of expression of the chromatin remodeling gene, SNF2L, selectively leads to DNA damage, growth inhibition, and cancer cell death. Mol Cancer Res.

[42] Ye, Y. *et al.**Singular v Dual Inhibition of SNF2L and Its Isoform, SNF2LT, Have Similar Effects on DNA Damage but Opposite Effects on the DNA Damage Response, Cancer Cell Growth Arrest and Apoptosis*. Vol. 3 (2012). 10.18632/oncotarget.479 PMC3380581 22577152

[43] Xu S (2015). The expression of AEG-1 and Cyclin D1 in human bladder urothelial carcinoma and their clinicopathological significance. Int J Clin Exp Med.

[44] Yang G (2014). AEG-1 is associated with tumor progression in nonmuscle-invasive bladder cancer. Med Oncol.

[45] Muppirala UK, Honavar VG, Dobbs D (2011). Predicting RNA-protein interactions using only sequence information. BMC Bioinformatics.

[46] Hedegaard, J. *et al.* Comprehensive Transcriptional Analysis of Early-Stage Urothelial Carcinoma. *Cancer Cell* In press, 10.1016/j.ccell.2016.05.004 (2016). 10.1016/j.ccell.2016.05.004 27321955

[47] van Heesch S (2014). Extensive localization of long noncoding RNAs to the cytosol and mono- and polyribosomal complexes. Genome Biol.

[48] Guttman M, Russell P, Ingolia NT, Weissman JS, Lander ES (2013). Ribosome profiling provides evidence that large noncoding RNAs do not encode proteins. Cell.

[49] Gupta RA (2010). Long non-coding RNA HOTAIR reprograms chromatin state to promote cancer metastasis. Nature.

[50] Yoon JH (2012). LincRNA-p21 suppresses target mRNA translation. Mol Cell.

[51] Nikpour M (2014). MTDH/AEG-1 contributes to central features of the neoplastic phenotype in bladder cancer. Urol Oncol.

[52] Meng X (2012). Cytoplasmic Metadherin (MTDH) provides survival advantage under conditions of stress by acting as RNA-binding protein. J Biol Chem.

[53] Willingham AT (2005). A strategy for probing the function of noncoding RNAs finds a repressor of NFAT. Science.

[54] Sarkar D, Fisher PB (2013). AEG-1/MTDH/LYRIC: clinical significance. Adv Cancer Res.

[55] Nordentoft I (2012). miRNAs associated with chemo-sensitivity in cell lines and in advanced bladder cancer. BMC Med Genomics.

[56] Ostenfeld MS (2010). miR-145 induces caspase-dependent and -independent cell death in urothelial cancer cell lines with targeting of an expression signature present in Ta bladder tumors. Oncogene.

[57] Christensen LL (2016). SNHG16 is regulated by the Wnt pathway in colorectal cancer and affects genes involved in lipid metabolism. Mol Oncol.

[58] Britze A, Birkler RI, Gregersen N, Ovesen T, Palmfeldt J (2014). Large-scale proteomics differentiates cholesteatoma from surrounding tissues and identifies novel proteins related to the pathogenesis. PLoS One.

[59] Cox J, Mann M (2008). MaxQuant enables high peptide identification rates, individualized p.p.b.-range mass accuracies and proteome-wide protein quantification. Nat Biotechnol.

[60] Damgaard CK, Lykke-Andersen J (2011). Translational coregulation of 5′TOP mRNAs by TIA-1 and TIAR. Genes Dev.

