# Supplementary material for: Profiling of long non-coding RNAs identifies LINC00958 and LINC01296 as candidate oncogenes in bladder cancer
Source: Sci Rep. 2017 Mar 24;7:395. doi: 10.1038/s41598-017-00327-0 (PMC5428251; doi:10.1038/s41598-017-00327-0)
Supplement: Supplementary file 1 — Supplementary INFO [file 41598_2017_327_MOESM1_ESM.pdf]

Profiling of long non-coding RNAs identifies LINC00958 and LINC01296 as candidate oncogenes in bladder cancer

Authors:

Anna Katharina Seitz[1,2,6], Lise Lotte Christensen[1,6], Emil Christensen[1], Kasper Faarkrog[1], Marie Stampe Ostenfeld[1], Jakob Hedegaard[1], Iver Nordentoft[1], Morten Muhlig Nielsen[1], Johan Palmfeldt[3], Michelle Thomson[3], Michael Theis Solgaard Jensen[4], Roman Nawroth[2], Tobias Maurer[2], Torben Falck Ørntoft[1], Jørgen Bjerggaard Jensen[5], Christian Kroun Damgaard[4] and Lars Dyrskjøt[1]*

Author Affiliations:

[1]Department of Molecular Medicine, Aarhus University Hospital, University of Aarhus, Aarhus, Denmark
[2]Department of Urology, Klinikum rechts der Isar, Technical University Munich, Munich, Germany
[3]Reserach unit for Molecular Medicine, Department of Clinical Medicine, Aarhus University Hospital, University of Aarhus, Aarhus, Denmark
[4]Department of Molecular Biology and Genetics - Genome expression, stability and technology, University of Aarhus, Aarhus, Denmark
[5]Department of Urology, Aarhus University Hospital, University of Aarhus, Aarhus, Denmark

[6]These authors contributed equally to this work and should be considered co-first authors

*Corresponding author: Lars Dyrskjøt (lars@clin.au.dk; +45 784 55320), Aarhus University Hospital, Skejby, Palle Juul-Jensens Boulevard 99, DK-8200, Aarhus N, Denmark, Fax +45 86782108

SUPPLEMENTARY MATERIALS

RT-qPCR primers (Sigma Aldrich, Denmark):

**Gen (human)**	**FORWARD (5'->3')**	**REVERSE (5'->3')**
LNC-ALX1-2	TCTTCTGTCAACCTGGTGCA	GTTGATTCAGGAACCCAGGG
LNC-CMC1-1	CACTGGATCCAAAGAGAGAAAC	AAATTGGCTGGCGGGTAG
LINC00355	TGGGTCTCCTCTGAGCTGTT	TGTCCTGTGTCCAGGATGAA
LINC00958	AGAGAGGAGGAGAAGCAA	TGTGAAGTGCAGGGAGGA
LINC01296	AACCCTCATCCATATCCT	TGGTTTTCTGGGTTTTGAC
MALAT1	CTTCCCTAGGGGATTTCAGG	GCCCACAGGAACAAGTCCTA
GAPDH	TGCACCACCAACTGCTTAGC	GGCATGGACTGTGGTCATGAG

LNA™ longRNA GapmeR *in vitro Premium* for knockdown of endogenous mRNA (Exiqon, Denmark):

**Gen (human)**	**LNA probe**	**Sequence (5'->3')**	**Exiqon ID**
Negative control A	Scr	AACACGTCTATACGC	300611-00
LNC-ALX1-2	LNA1	ATCATTAAGGATGGCA	314916-1
LNC-ALX1-2	LNA2	CTGATAGAAGTGAATT	314916-2
LNC-CMC1-1	LNA1	ACCTAGATCATAATTC	361213-1
LNC-CMC1-1	LNA2	CCTAGATCATAATTCT	361213-2
LINC00355	LNA1	GCGAGTAAGCAGAAGA	361192-1
LINC00355	LNA2	GTAAGGCAGACAAATG	361192-2
LINC00958	LNA1	TCGAGGCAATGATTAA	302216-1
LINC00958	LNA2	AGCAGTGATCGAGGCA	302216-2
LINC01296	LNA1	TGTGGAAGGAAGAGTC	302295-2
LINC01296	LNA2	TGATTGACAGATGACA	302295-1

Cloning primers containing restriction site (Sigma Aldrich, Denmark):

Gen (human)	Transcript ID	FORWARD (5'->3')	REVERSE (5'->3')
LINC00958	ENST00000534477.1	ATACGAATTCTCTCTCCTGCTGCATTGTG	TATGCTCGAGACACACACTCCAGCAAAG
LINC01296	ENST00000400192.4	TACAGTCGACGAGCTTCGGAGAAGCAGT	TACACTCGAGGGGCATCTTGGAGTGAGA

Restriction sites (underlined): EcoRI/XhoI (LINC00958) and SalI/Xho1 (LINC01296)

Sequencing primers (TOPO® TA Cloning® Kit for Sequencing**,** Life Technologies, Carlsbad, USA):

Name	Sequence (5'->3')
T3 primer	ATTAACCCTCACTAAAGGGA
T7 primer	TAATACGACTCACTATAGGG

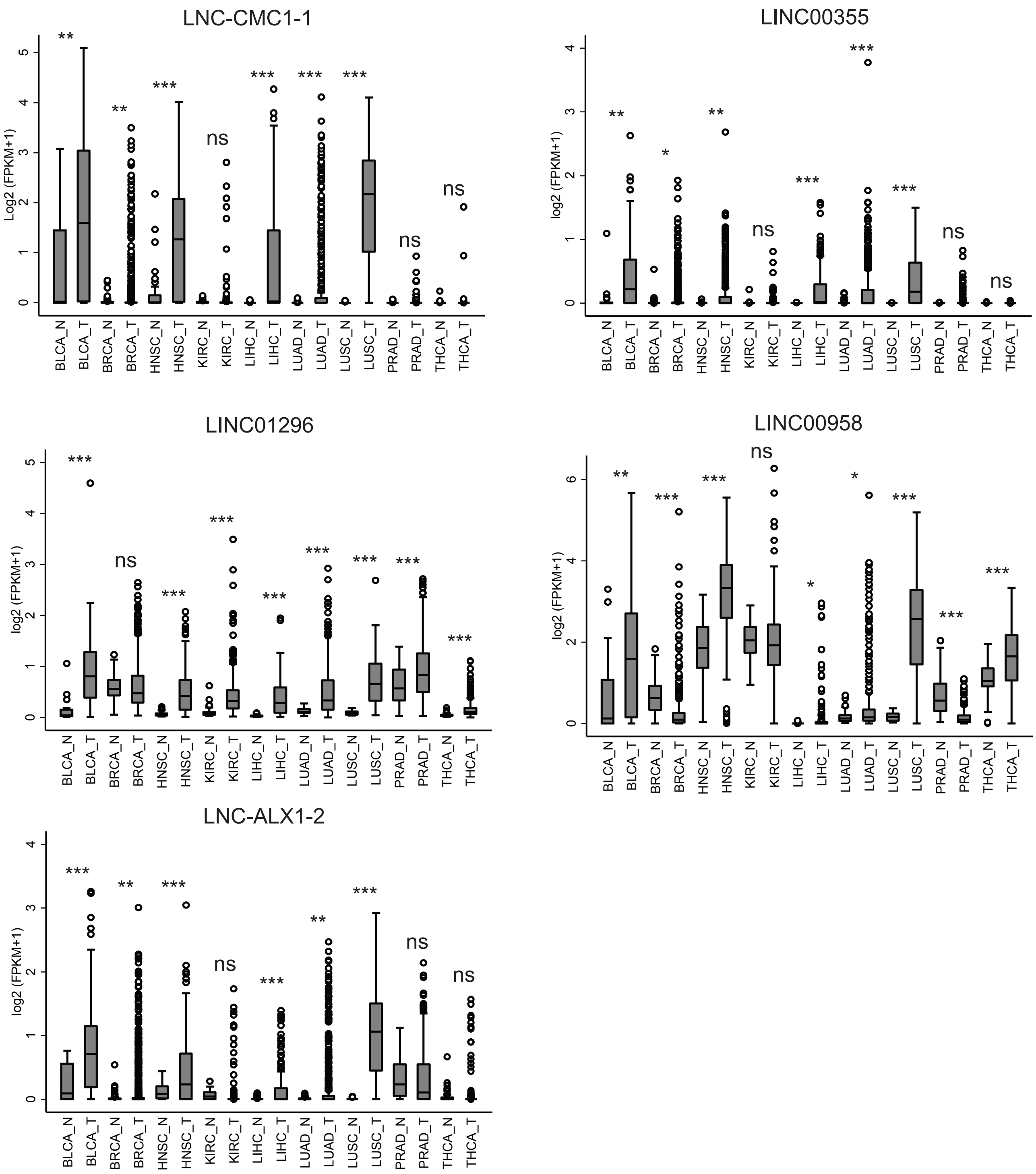

Supplementary Figure S1. Expression of the five selected lincRNAs based on data from TCGA and downloaded from Tanric. BLCA: bladder urothelial carcinoma (n=19 (N) and n=252 (T)), BRCA: breast invasive carcinoma (n=105 (N) and n=837 (T)), HNSC: head and neck squamous carcinoma (n=42 (N) and n=426(T)), KIRC: kidney renal clear cell carcinoma (n=67 (N) and n=448 (T)), LIHC: liver hepatocellular carcinoma (n=50 (N) and n=200 (T)), LUAD: lung adenocarcinma (n=58 (N) and n=488 (T)), LUSC: lung squamous cell carcinoma (n=17 (N) and n=220 (T)), PRAD: prostate adenocarcinoma (n=52 (N) and n=374 (T)) and THVA: thyroid carcinoma (n=59 (N) and n=497 (T)). N: normal, T: tumor and NS: not signicant. *$p < 0.05$, **$p < 0.01$ and ***$p < 0.001$ (Student's *t*-test).

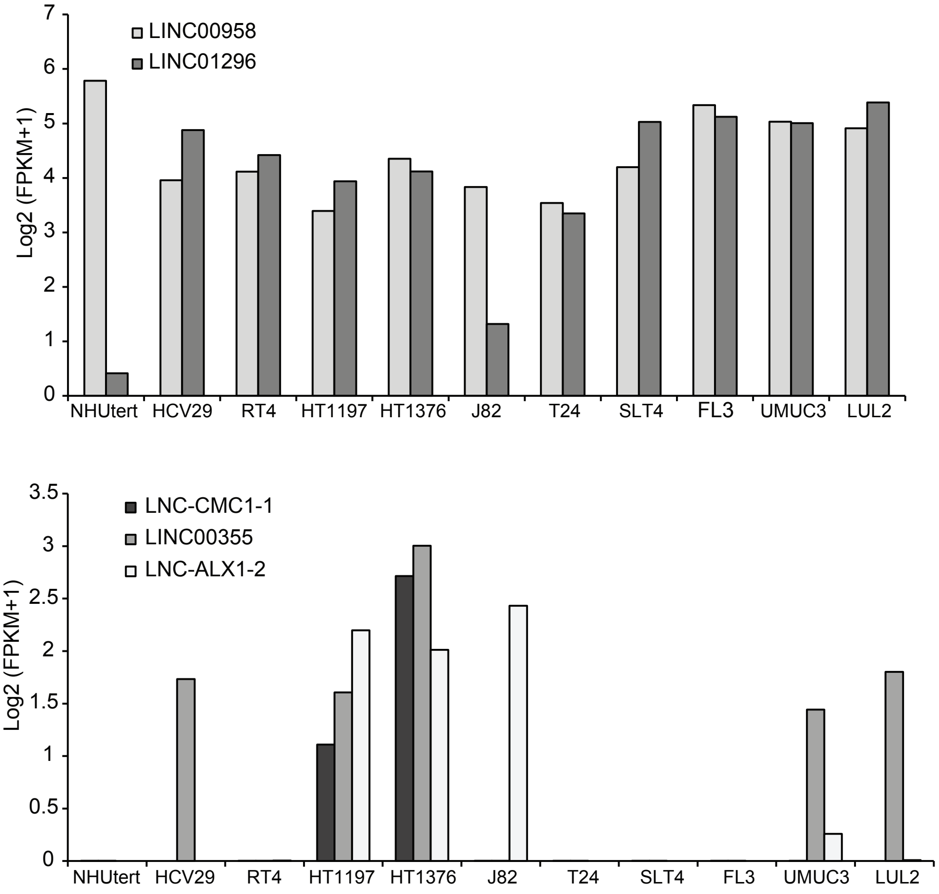

Supplementary Figure S2. **Expression of selected lncRNA candidatates in 11 bladder cancer cell lines**. The expression was measured using RNA-seq.

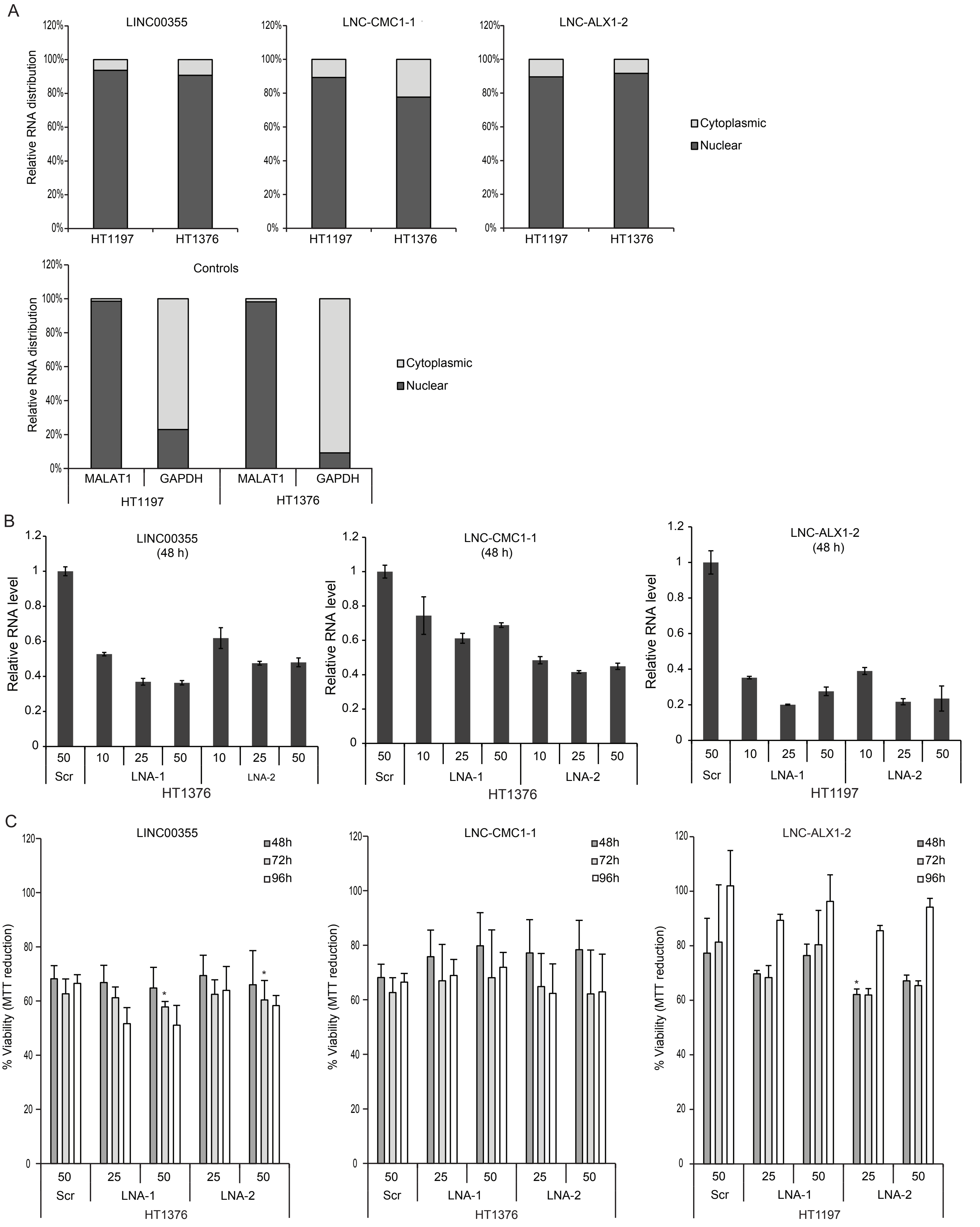

Supplementary Figure S3. Subcellular distribution, knockdown efficiency and functional impact of LINC00355, LNC-CMC1-1 and LNC-ALX1-2 in HT1376 and HT1197 cells. (A) Percentage of total RNA found in the nuclear and cytoplasmic fraction of HT1376 and HT1197 cells. Upper panel: LincRNA-candidates. Lower panel: Nuclear (MALAT1) and cytoplasmic (GAPDH) markers confirming fraction purity. (B and C) HT1376 and HT1197 cells were transfected with a non-targeting LNA (Scr) or 2 different LNAs targeting either LINC00355, LNC-CMC1-1 or LNC-ALX1-2 (LNA-1 and LNA-2) at the indicated concentrations (nM). (B) Dose-dependent knockdown efficiency was verified by RT-qPCR 48 hours post-transfection. (C) Dose-dependent viability was assessed by MTT reduction 48, 72 and 96 h post-transfection and expressed as percentage compared to viability of untransfected cells. Columns in A and B represent a single experiment measured in three replicates and shown as the mean ± SD. Columns in C, average of two independent experiments, each measured in three replicates. Bars: SD and *p<0.05.

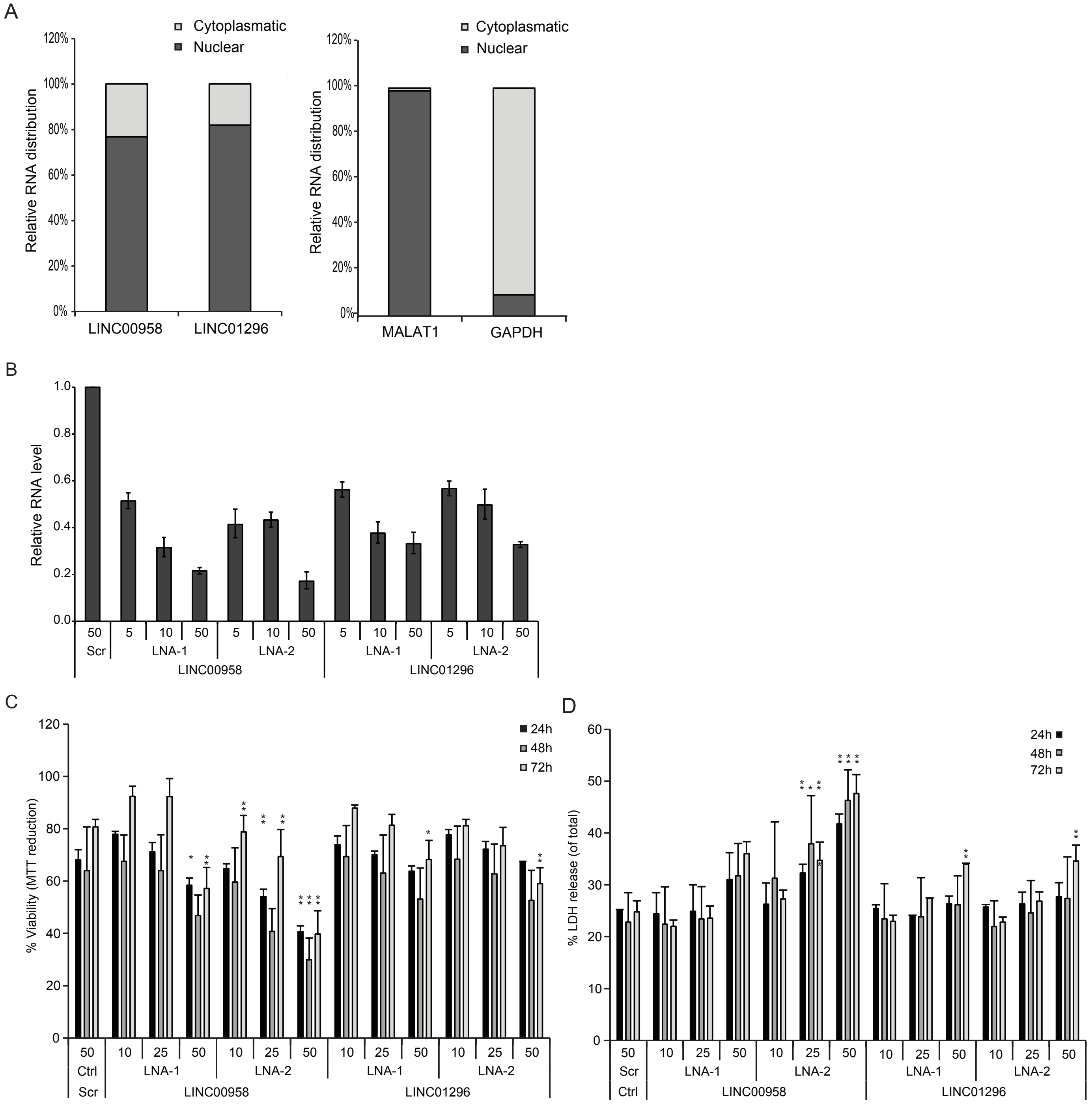

Supplementary Figure S4. Functional effects of LINC00958 or LINC01296 knockdown in RT4 cells. (A) Percentage of total RNA found in the nuclear and cytoplasmic fraction of RT4 cells. Left panel: LincRNA-candidates. Right panel: Nuclear (MALAT1) and cytoplasmic (GAPDH) markers confirming fraction purity. (B-D) RT4 cells were transfected either with a non-targeting LNA™GapmeR (Scr) or 2 different LNA™GapmeR separately targeting LINC00958 or LINC01296 (LNA-1 and LNA-2) at the indicated concentrations (mM). (B) Dose-dependent knockdown efficiency was verified by RT-qPCR 48 h post-transfection. Results were normalized to Scr. (C) Dose-dependent viability was assessed by MTT reduction 24, 48 and 72 h post-transfection and expressed as percentage compared to viability of untransfected cells. (D) Dose-dependent cell death was determined by the LDH release assay 24, 48 and 72 h post-transfection and depicted as percentage of released LDH out of total cellular LDH. Columns in A and B represent a single experiment measured in three replicates and shown as the mean ± SD. Columns in C represents the average of two or three independent experiments, each measured in three to five replicates. Bars: SD and p<0.05 and **p<0.01.

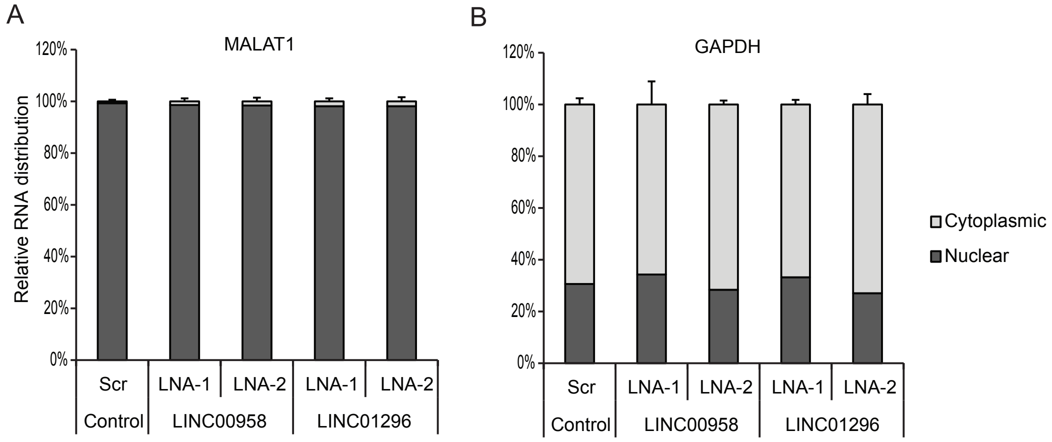

Supplementary Figure S5. Confirmation of RNA fraction purity in LNAGapmeR transfected FL3 cells. (A and B) FL3 cells were transfected with the indicated LNAs (50 nM) followed by RNA fractionation 48 hours post-transfection. RNA fraction purity was verified by a control genes whose transcripts were roughly exclusively present either in the nucleus or the cytoplasm. Relative RNA distribution was determined by RT-qPCR and is shown as percentage of total RNA. A, nuclear marker. B, Cytoplasmic marker. Columns in A and B, average of two independent experiments, each measured in three replicates. Bars: SD.

A

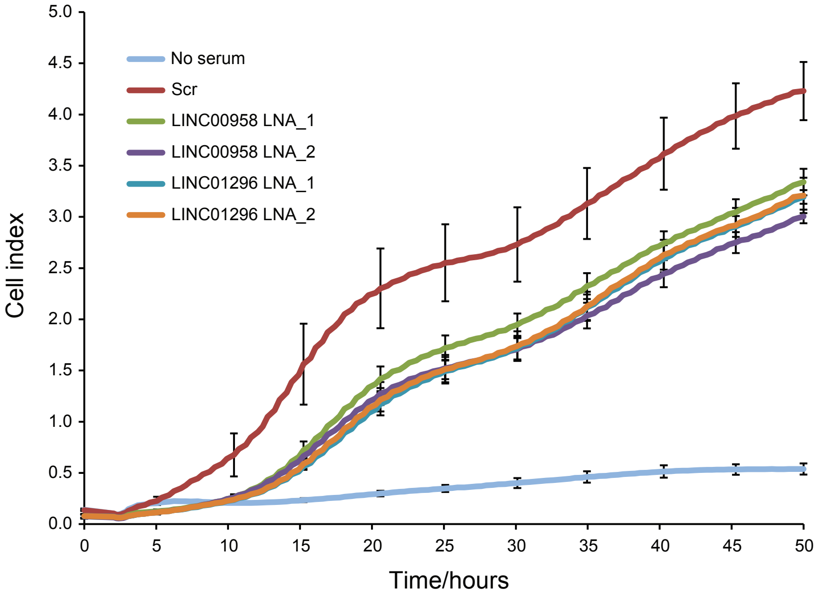

B

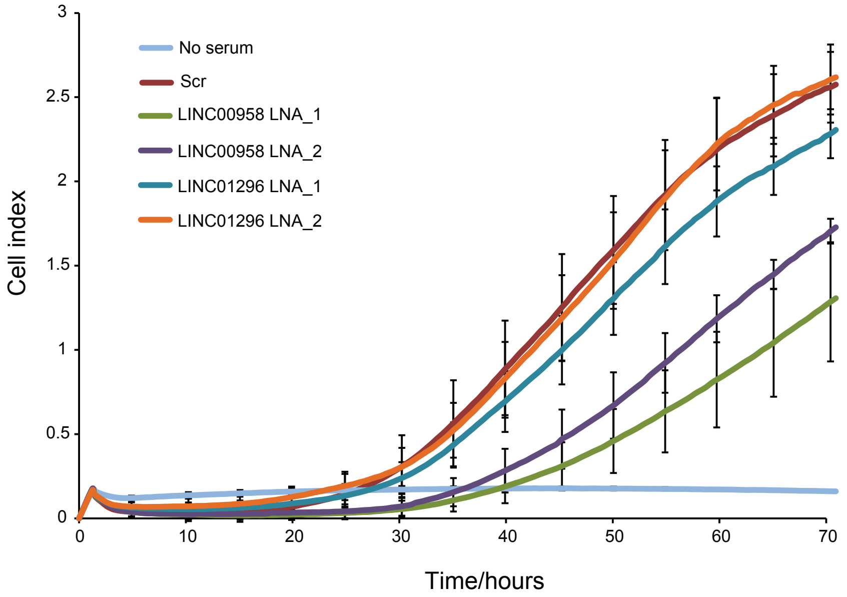

Supplementary Figure S6. Real-time monitoring of cell migration (A) and invasion (B). FL3 cells were transfected with 25 nM LNAGapMer LNA_1 or LNA_2 and real time monitoring was performed using an xCELLigence instrument. The cell index measured in 3-5 replicates ± SD from time 0-50 (migration) or 0-70 (invasion) hours is shown.

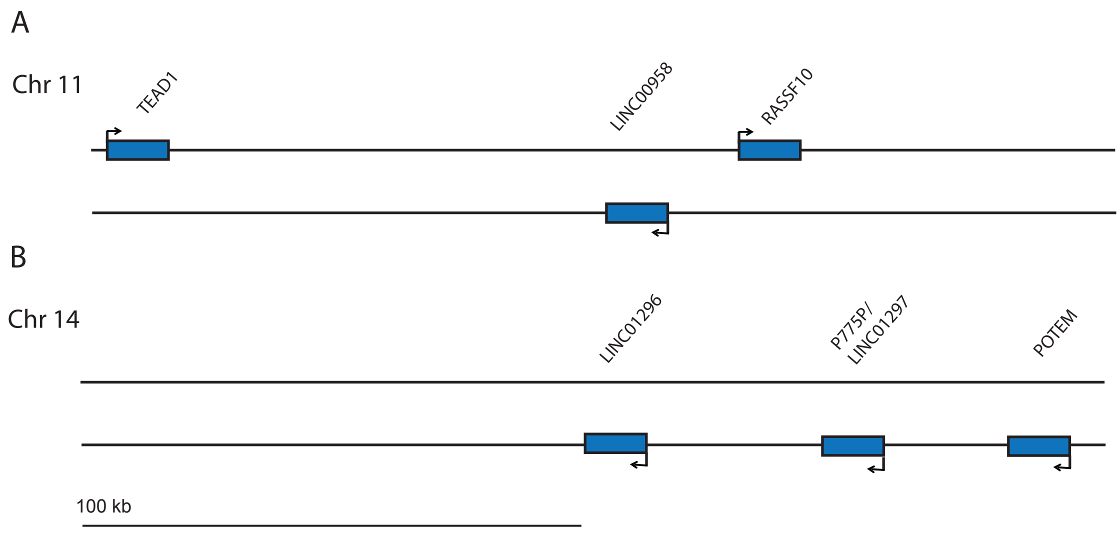

Supplementary Figure S7. Physical map of LINC00958 (A) and LINC01296 (B). Shown is the lncRNA locus and the surrounding genes within 100 kbs up- or downstream (Hg19). The expression of LINC0958 was not correlated to TEAD1 (Spearman's rho 0.20 and p=0.09) whereas the expression of LINC01296 was significantly correlated to both LINC01297 (Spearman's rho 0.60 and p<0.0001) and POTEM (Spearman's rho 0.48 and p<0.0001). The correlation between RASSF10 and LINC00958 was not analyzed (no data on RSSF10 expression).

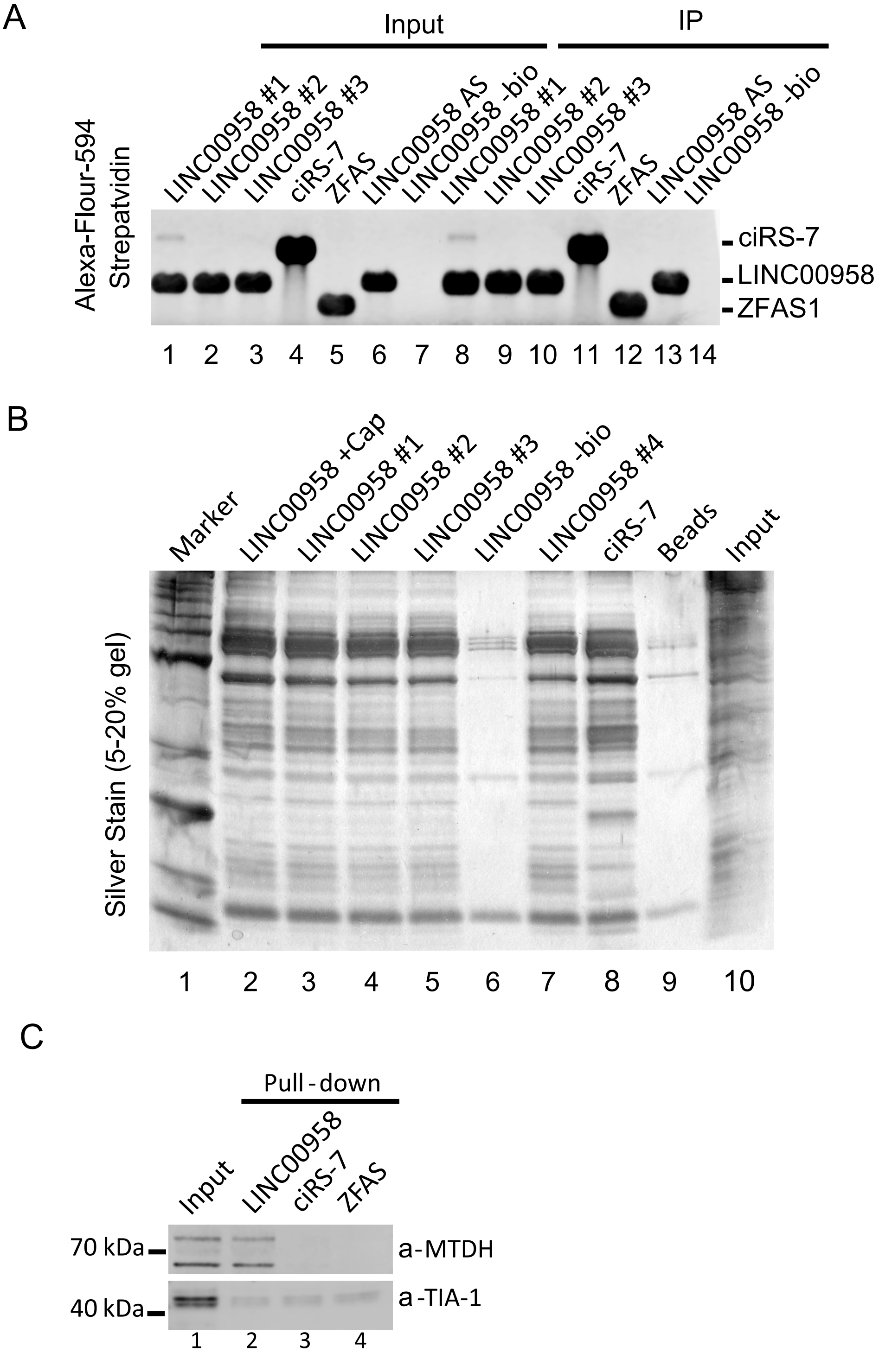

Supplementary Figure S8. **Control experiments RNA pull down analysis.** A) Northern blot of in vitro-transcribed and biotinylated RNAs (Inputs and pull-down fractions). Approximately 5 µg input RNA and 25% of pulled down RNA was detected by Alexa594-conjugated Streptavidin and visualized using a Licor OdysseyFc scanner. B) Representative silver-stained SDS gradient gel (5-20%) from LINC00958 pull-down experiment, (4 replicates), unrelated RNA (ciRS-7), bead- and non-biotinylted RNA control. C) Western blot from pull-down showing that MTDH is specifically captured by LINC00958.

Supplementary Table S1. Coding-Potential Assessment Tool (CPAT) and PhastCons 46-way conservation track for key lncRNA-candidates.

Transcript name	Transcript ID	RNA size (nt)	Coding Probability	Coding Label	Known status	PhastCons score
LINC00958	ENST00000527945.1	453	0.055458917	no	non-coding	0
	ENST00000504230.2	1558	0.010092033	no	non-coding	0.0237
	ENST00000532541.1	1124	0.01035872	no	non-coding	0.0632
	ENST00000534477.1	888	0.010506654	no	non-coding	0.08
	ENST00000529328.1	580	0.033117461	no	non-coding	0
	ENST00000531402.1	590	0.020176496	no	non-coding	0.0576
	ENST00000526388.1	607	0.0201561	no	non-coding	0.056
LINC01296	ENST00000551334.1	3426	0.285358583	no	Non-coding	0.4
	ENST00000546382.1	649	0.019252136	no	non-coding	0
	ENST00000552602.1	4264	0.00935778	no	non-coding	0.0197
	ENST00000553153.1	903	0.010002305	no	non-coding	0
	ENST00000455088.2	2397	0.010469297	no	non-coding	0.035
	ENST00000549813.1	1013	0.01883923	no	non-coding	0.0079
	ENST00000547648.1	549	0.042017289	no	non-coding	0
	ENST00000547285.1	1403	0.021208628	no	non-coding	0
	ENST00000550805.1	631	0.003558891	no	non-coding	0
	ENST00000548875.1	412	0.005113057	no	non-coding	0
	ENST00000400192.4	996	0.024901232	no	non-coding	0
	ENST00000548903.1	576	0.019336006	no	non-coding	0
	ENST00000548057.2	1445	0.00331922	no	non-coding	0.558
	ENST00000546959.1	413	0.004610957	no	non-coding	0
	ENST00000549065.1	605	0.019302645	no	non-coding	0
LINC00355	ENST00000456627.1	1878	0.039463949	no	non-coding	0
LNC-CMC1-1	ENST00000425195.1	491	0.019712318	no	non-coding	0
	ENST00000414382.1	543	0.047079178	no	non-coding	0
	ENST00000455984.1	332	0.028494737	no	non-coding	0
LNC-ALX2-1	ENST00000555596.1	519	0.024282657	no	non-coding	0
MALAT1*	ENST00000534336.1	8708	0.014255836	no	non-coding	-†
	ENST00000544868.1	4585	0.019288203	no	non-coding	-
	ENST00000508832.1	1031	0.002221975	no	non-coding	-
GAPDH*	ENST00000229239.5	1875	0.999962146	yes	coding	-
	ENST00000396856.1	1266	0.999679465	yes	coding	-
	ENST00000492719.1	930	0.997496149	yes	coding	-
	ENST00000396861.1	1348	0.999963338	yes	coding	-
	ENST00000474249.1	1333	0.999794938	yes	coding	-
	ENST00000466588.1	1363	0.999794564	yes	coding	-
	ENST00000396859.1	1256	0.999963542	yes	coding	-
	ENST00000466525.1	1720	0.994275134	yes	coding	-
	ENST00000396858.1	1292	0.999870952	yes	coding	-
	ENST00000509325.1	1014	0.841509089	yes	coding	-

*Known lncRNA MALAT1 and protein coding gene GAPDH were included as positive controls.

†Not analysed

Supplementary Table S2. mRNAs significantly dys-regulated in FL3 cells transfected with GapmeRs targeting LINC00958 and correlated to LINC00958 in clinical samples (n=72).

Gene id	IPA Pathway*	FC (log2) siRNA KD	r_s Clinical samples
**AIG1***	-	**-0.96**	**0.6**
C20orf194	-	-0.69	0.5
GTF2IRD1	-	-0.67	0.5
PLEKHA2	-	-0.61	0.5
SLC16A7	-	-0.92	0.5
CRYZ	-	-0.83	0.5
CAST	CM/CDS	-0.93	0.4
CASD1	-	-1.00	0.5
TMTC2	-	-1.10	0.5
PP1R9A	-	1.00	0.5
SBF2	-	-1.30	0.5
RCAN3	-	0.62	0.4
LANCL1	-	-0.64	0.5
NSUN4	-	-0.73	0.4
MCCC2	SMB	-0.65	0.4
GPR126	-	-0.86	0.4
RNLS	-	-1.38	0.5
NTN4	-	-0.65	0.4
TES	CGP	-0.78	0.4
PIGB	-	-0.70	0.4
TRIM5	-	-0.64	0.4
ZMIZ1	-	-0.87	0.4
FBXO36	-	-0.82	0.5
NINL	-	-0.60	0.4
BBS2	-	-1.00	0.4
KIAA1324L	-	-0.94	0.4
UST	-	-0.67	0.4
ZSWIM6	-	-0.98	0.4
**SMARCA1**	**CCS/CDS/CGP**	**-0.70**	**0.4**
**ITGA3**	**CCS/CDS/CM/SMB**	**-0.77**	**0.4**
USP47	CDS	-1.14	0.4
VPS41	-	-0.80	0.4
MSH3	-	-0.88	0.4
LMBRD1	-	-0.98	0.4
TMC7	-	-0.70	0.4
ARIH1	-	-1.13	0.4
PTPRS	-	-0.73	0.4
PTPN9	-	-0.42	0.4
UBE3A	CGP	-0.77	0.5
BCKDHB	-	-1.27	0.4
NXN	-	-0.80	0.5
ARID1B	-	-0.69	0.4
KIF13B	-	-0.68	0.4
H2AFY2		-0.79	0.4

Selection criteria:

1) Genes down-regulated upon LINC00958 KD ($FC_{(log2)}$ >-0.6, p< 0.05) and positively correlated to LINC00958 (Spearman's ρ (r_s) ≥0.4and p<0.01) in clinical samples (44 genes identified)

2) Genes up-regulated upon LINC00958 KD ($FC_{(log2)}$ >0.6, p< 0.05) and negatively correlated to LINC00958 (Spearman's ρ (r_s) ≤-0.4 and p<0.01) in clinical samples (none identified)

*Genes that were correlated to LINC00958 in an independent sample set (n = 476) (Spearman's ρ (r_s)≥0.4 and p<0.01) are in bold.

CM: Cellular Movement, CDS: Cell Death and Survival, SMB: Small Molecule Biochemistry, CGP: Cellular Growth and Proliferation and CCS: Cell to Cell Signaling.

Supplementary Table S3. mRNAs significantly dys-regulated in FL3 cells transfected with GapmeRs targeting LINC01296 and correlated to LINC01296 in clinical samples (n=72).

Gene id	IPA Pathway*	FC (log2) siRNA KD	r_s Clinical samples
EXTL2	-	-0.80	0.4
CCDC14	-	-0.64	0.4
DIAPH3	-	-1.56	0.4
WDPCP	-	-0.61	0.4
RFC3	-	-0.64	0.4
NBAS	-	-1.37	0.4
RNF24	-	-0.85	0.4
LARP4B	-	-0.77	0.4
ZC4H2	-	-0.66	0.4
SRPK2	CGP	-0.65	0.4
INADL	-	-0.73	0.4
ZMYM2	CGP/CDS	-0.67	0.4
TFCP2	CDS/GE/GD	-0.73	0.4
USP6NL	-	-1.10	0.4
ZC2HC1A	-	-1.21	0.4
THADA	-	-0.81	0.4
ARID2	GD	-1.63	0.4
C20orf96	-	-0.61	0.4
VPS13B	-	-0.75	0.5
PLCG1	-	-1.06	0.5
SLC44A5	-	-0.89	0.5
DPY19L3	-	-0.69	0.5
C6orf170	-	-0.83	0.5
HOOK1	-	-0.86	0.5
EXOC4	-	-0.87	0.5

Selection criteria:

1) Genes down-regulated upon LINC01296 ($FC_{(log2)}$ >-0.6, $p < 0.05$) and positively correlated to LINC01296 (Spearman's ρ (r_s) ≥0.4and p<0.01) in clinical samples (25 genes identified)

2) Genes up-regulated upon LINC01296 KD ($FC_{(log2)}$ >0.6, $p < 0.05$) and negatively correlated to LINC01296 (Spearman's ρ (r_s) ≤-0.4 and p<0.01) in clinical samples (none identified)

*Genes that were correlated to LINC01296 in an independent sample set (n = 476) (Spearman's ρ (r_s)≥0.4 and p<0.01) are in bold.

CDS: Cell Death and Survival, CGP: Cellular Growth and Proliferation, GD: Gastrointestinal Disease and GE: Gene Expression

Supplementary Table S4. Proteins from LINC00958 in vitro pull-down and MS analyses associated with "Canonical Pathways" (IPA analyses)*

Gene id	FC MS	Function	Cellular localization
AGO2	9.78	Translation regulator	Cytoplasmic
EIF3A	10.33	Translation initiation	Cytoplasmic
EIF3D	13.13	Translation initiation	Cytoplasmic
EIF3F	14.13	Translation initiation	Cytoplasmic
EIF3L	17.14	Translation initiation	Cytoplasmic
EIF4G1	50.98	Translation initiation	Cytoplasmic
FAU	7.45	Ribosomal protein	Cytoplasmic
PABPC1	19.92	Translation regulator	Cytoplasmic
PPP1CA	4.29	Translation regulator	Cytoplasmic
RPL10	623.33	Ribosomal protein	Cytoplasmic
RPL10A	16.94	Ribosomal protein	Nuclear
RPL13	10.82	Ribosomal protein	Nuclear
RPL3	7.84	Ribosomal protein	Nuclear
RPL31	319.14	Ribosomal protein	Cytoplasmic
RPL4	8.39	Ribosomal protein	Cytoplasmic
RPL6	20.33	Ribosomal protein	Nuclear
RPL8	3.15	Ribosomal protein	-
RPS16	92.3	Ribosomal protein	Cytoplasmic
RPS2	10.71	Ribosomal protein	Cytoplasmic
RPS3	7.10	Ribosomal protein	Cytoplasmic
RPS3A	11.46	Ribosomal protein	Nuclear
RPS4X	6.74	Ribosomal protein	Cytoplasmic
RPS6	10.76	Ribosomal protein	Cytoplasmic
RPS7	1666.53	Ribosomal protein	Cytoplasmic
RPS8	32.02	Ribosomal protein	Cytoplasmic
RPS9	506.37	Ribosomal protein	Cytoplasmic

*Proteins associated with the following pathways are shown: EIF2 signaling, regulation of eIEF4 and p70S6K signaling and/or mTOR signaling

Supplementary Table S5. Proteins from LINC00958 in vitro pull-down and MS analyses associated with "Diseases and Functions" (IPA analyses)*

Gene id	FC MS	Function	Cellular localization
BCAS2	22.69	RNA processing	Nuclear
C1QBP	10.04	Transcription regulator	Cytoplasmic
CASC3	90.50	RNA processing	Nuclear
DDX20	9.80	RNA secondary structure	Nuclear
DDX21	9.59	RNA secondary structure	Nuclear
DDX47	7.48	RNA secondary structure	Nuclear
DDX5	5.09	RNA secondary structure	Nuclear
FBL	10.07	RNA processing	Nuclear
HNRNPAO	3.92	RNA processing	Nuclear
HNRNPF	6.54	RNA processing	Nuclear
HNRNPH1	6.68	RNA processing	Nuclear
HNRNPM	5.11	RNA processing	Nuclear
MBNL1	5.29	RNA processing	Nuclear
NPM1	8.39	Transcription regulator	Nuclear
PABPC1	10.92	Translation regulator	Cytoplasmic
PABPC4	10.61	Translation regulator	Cytoplasmic
PES1	10.56	RNA processing	Nuclear
PRPF4	3.07	RNA processing	Nuclear
RBMS1	3.97	RNA processing	Nuclear
RPS16	92.3	Ribosomal protein	Cytoplasmic
RPS6	10.76	Ribosomal protein	Cytoplasmic
RPS7	1666.53	Ribosomal protein	Cytoplasmic
SART3	8.08	RNA processing	Nuclear
SFPQ	6.29	RNA processing	Nuclear
SRPK1	15.83	RNA processing	Nuclear
SRSF9	127.63	RNA processing	Nuclear
SSB	7.96	RNA processing	Nuclear
TARDBP	16.53	RNA processing	Nuclear
THRAP3	4.86	RNA processing	Nuclear
WDR43	11.67	RNA processing	Nuclear
WDR75	10.11	RNA processing	Nuclear
WTAP	16.00	RNA processing	Nuclear
YTHDF2	15.48	RNA stability	-
ZNF326	7.05	Transcription regulator	Nuclear

*Proteins associated with the biological function "RNA post-transcriptional modifications" are shown.

Supplementary Table S6. Top30 proteins binding LINC00958 in the RNA pull-down assay

Protein name	Gene name	FC (LINC00958 vs controls)
40S ribosomal protein S17-like;40S ribosomal protein S17	RPS17L;RPS17	-*
40S ribosomal protein S13	RPS13	-
Periodic tryptophan protein 1 homolog	PWP1	-
Flotillin-2	FLOT2	-
Nucleolar MIF4G domain-containing protein 1	NOM1	-
DnaJ homolog subfamily C member 21	DNAJC21	-
RRP12-like protein	RRP12	-
Ubiquitin-associated protein 2	UBAP2	-
Protein virilizer homolog	KIAA1429	-
CLIP-associating protein 1	CLASP1	-
Protein LYRIC	MTDH	-
Zinc finger CCCH domain-containing protein 7A	ZC3H7A	-
Plasminogen activator inhibitor 1 RNA-binding protein	SERBP1	-
ATP-dependent RNA helicase DDX55	DDX55	-
Zinc finger CCHC-type and RNA-binding motif-containing protein 1	ZCRB1	-
Selenocysteine insertion sequence-binding protein 2-like	SECISBP2L	-
Twinkle protein, mitochondrial	PEO1	-
60S ribosomal protein L27	RPL27	-
Borealin	CDCA8	-
40S ribosomal protein S7	RPS7	2210.3
40S ribosomal protein S11	RPS11	1296.0
60S ribosomal protein L10	RPL10	822.7
40S ribosomal protein S9	RPS9	665.5
60S ribosomal protein L17	RPL17	460.4
60S ribosomal protein L31	RPL31	422.1
40S ribosomal protein S25	RPS25	412.2
40S ribosomal protein S23	RPS23	375.9
7SK snRNA methylphosphate capping enzyme	MEPCE	295.4
60S ribosomal protein L24	RPL24	184.4

*-: no binding in controls
